# Transcranial focused ultrasound modulates cortical and thalamic motor activity in awake sheep

**DOI:** 10.1038/s41598-021-98920-x

**Published:** 2021-09-29

**Authors:** Hyun-Chul Kim, Wonhye Lee, Jennifer Kunes, Kyungho Yoon, Ji Eun Lee, Lori Foley, Kavin Kowsari, Seung-Schik Yoo

**Affiliations:** 1 Department of Radiology, Brigham and Women’s Hospital, Harvard Medical School, 75 Francis Street, Boston, MA 02115 USA; 2 Translational Discovery Laboratory, Brigham and Women’s Hospital, Boston, MA USA; 3 Department of Mechanical Engineering, Massachusetts Institute of Technology, Cambridge, MA USA

**Keywords:** Electromyography - EMG, Motor control

## Abstract

Transcranial application of pulsed low-intensity focused ultrasound (FUS) modulates the excitability of region-specific brain areas, and anesthetic confounders on brain activity warrant the evaluation of the technique in awake animals. We examined the neuromodulatory effects of FUS in unanesthetized sheep by developing a custom-fit headgear capable of reproducibly placing an acoustic focus on the unilateral motor cortex (M1) and corresponding thalamic area. The efferent responses to sonication, based on the acoustic parameters previously identified in anesthetized sheep, were measured using electromyography (EMG) from both hind limbs across three experimental conditions: on-target sonication, off-target sonication, and without sonication. Excitatory sonication yielded greater amplitude of EMG signals obtained from the hind limb contralateral to sonication than that from the ipsilateral limb. Spurious appearance of motion-related EMG signals limited the amount of analyzed data (~ 10% selection of acquired data) during excitatory sonication, and the averaged EMG response rates elicited by the M1 and thalamic stimulations were 7.5 ± 1.4% and 6.7 ± 1.5%, respectively. Suppressive sonication, while sheep walked on the treadmill, temporarily reduced the EMG amplitude from the limb contralateral to sonication. No significant change was found in the EMG amplitudes during the off-target sonication. Behavioral observation throughout the study and histological analysis showed no sign of brain tissue damage caused by the acoustic stimulation. Marginal response rates observed during excitatory sonication call for technical refinement to reduce motion artifacts during EMG acquisitions as well as acoustic aberration correction schemes to improve spatial accuracy of sonication. Yet, our results indicate that low-intensity FUS modulated the excitability of regional brain tissues reversibly and safely in awake sheep, supporting its potential in theragnostic applications.

## Introduction

Over the past decade, the exquisite spatial specificity and deep penetration depth of transcranial focused ultrasound (tFUS) have drawn significant attention from the scientific community as a new non-invasive brain stimulation modality. The pulsed application of low-intensity FUS has been shown to reversibly modulate (either activate or suppress) region-specific brain excitability in small animal models, such as rabbits^1^ and rodents^2–6^. Further studies have validated the neuromodulatory effects of FUS among large animals^7^ and non-human primates^8,9^. We previously demonstrated the bimodal (*i.e.*, excitatory and suppressive) neuromodulatory effects of FUS to the sensorimotor cortical and thalamic pathway in anesthetized sheep^10,11^. The ovine model provides rich translational information regarding neuromodulatory FUS due to the similarities in cranial and neuroanatomical features to those of humans^12,13^. Availability of neuropathological models, such as Alzheimer’s disease^14^, epilepsy^15^, and stroke^16^, also renders sheep as an attractive species for investigation.

In many animal studies that probed the neuromodulatory effects of tFUS, anesthesia has been utilized. As anesthetic agents generally affect the state of brain tissue excitability^17^, anesthesia is recognized as one of the confounding factors that may alter neural response to acoustic stimulation of the brain^5,11,18–20^. For example, the types of agents (*e.g.*, isoflurane or ketamine/xylazine) and their dose heavily influenced the degree of responses to the stimulations^18,20^. In order to examine the effects of acoustic brain stimulation in the absence of the confounding effects from anesthesia, we were motivated to apply low-intensity FUS to the brain of unanesthetized, freely moving sheep.

While non-human primates can be trained to immobilize their head (often using a head fixation apparatus) for the application of FUS, reproducible and precise sonication to a desired brain location is a challenging task when using awake sheep. To address this challenge, we initially implanted a plastic pedestal onto the skull that mounted a FUS transducer, but the implant failed to perform due to mechanical instability. In the present study, as an alternative to the surgical approach, we developed 3D-printed FUS transducer headgear that can be worn on the sheep’s head in a spatially reproducible manner. The headgear contained magnetic resonance imaging (MRI)-visible fiducial markers and was positioned with respect to three tattoo markings made on the sheep’s scalp, allowing for reproducible administration of FUS in awake sheep while they either stayed still (for excitation) or walked on a treadmill (for suppression).

Anatomical and functional MRI-based navigation provided guidance on sonicating the unilateral primary motor cortical (M1) and the thalamic areas while electromyography (EMG) was acquired wirelessly from both hind limbs to evaluate the efferent outcome of the FUS stimulation of the brain by measuring changes in EMG amplitude obtained across three experimental conditions—sonication of the target, sonication of an off-target non-motor area, and without sonication. We report similarities and differences in the neuromodulatory outcomes to the ones previously demonstrated in anesthetized sheep, along with a safety evaluation through behavioral monitoring and histological assessment.

## Results

### Wearable FUS headgear and image-guided FUS

**Figure 1:**
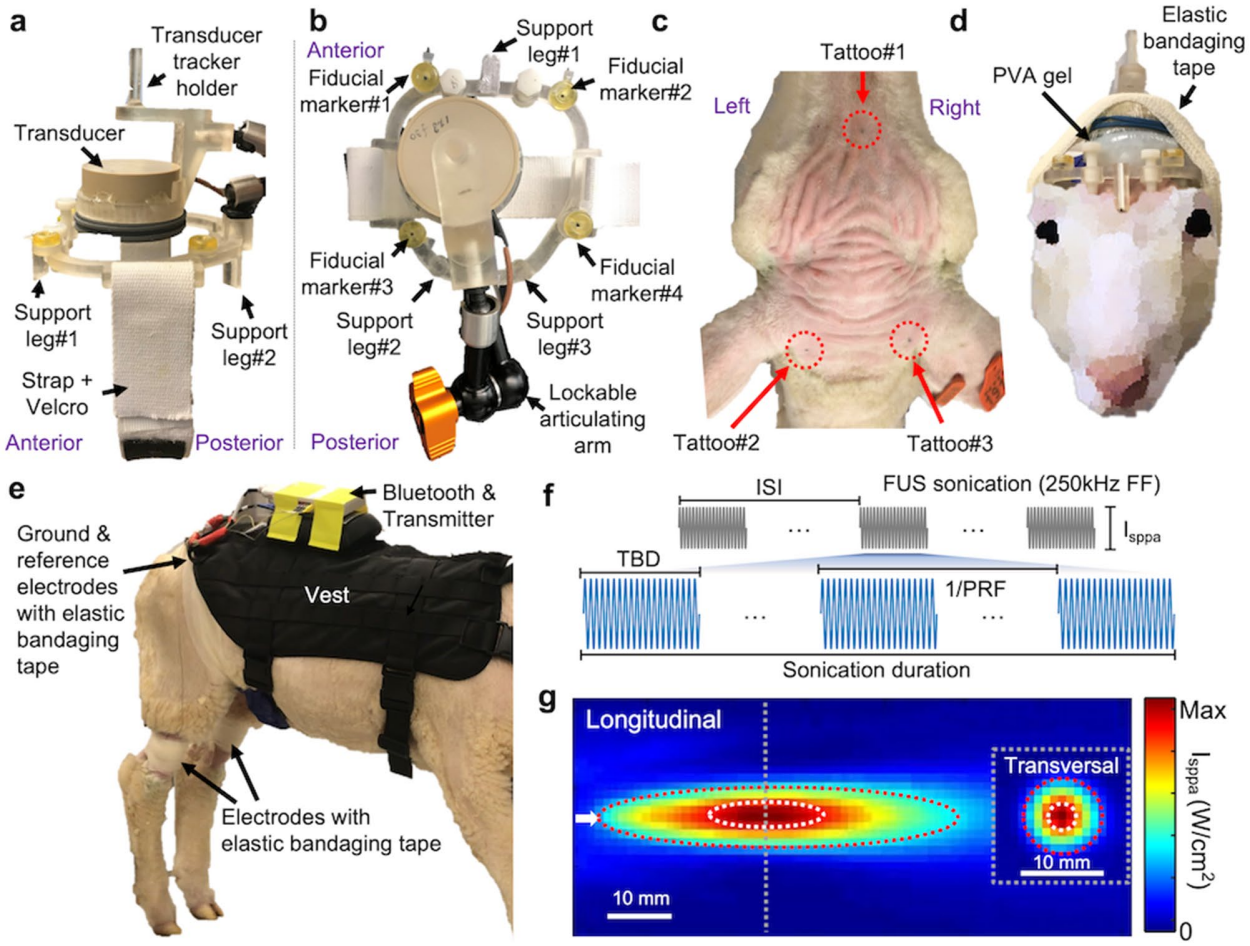
Schematics of the experimental setup. Wearable FUS headgear viewed **(a)** from the side and (**b**) from the top. (**c**) Exemplary tattoo locations on the sheep’s scalp, which were used for reproducible positioning of the headgear. (**d**) An illustration of the headgear with the acoustic-coupling polyvinyl alcohol (PVA) hydrogel worn by sheep (secured with elastic bandaging tape). (**e**) An experimental setup for EMG acquisition. A vest was used to attach a telemetry EMG Bluetooth transmitter and preamplifier. The ground and reference electrodes were placed on the back, and an electrode was placed on the gastrocnemii of each hind limb. All electrodes were secured using elastic tape. (**f**) An illustration of acoustic parameters. (**g**) An acoustic intensity profile across the longitudinal and transversal planes (dotted gray line). Dotted white lines depict the regions of 90%-maximum of the intensity whereas dotted red lines depict the regions of full-width at half-maximum of the intensity. The white arrow represents the sonication direction. FF, fundamental frequency; I_sppa_, spatial-peak pulse-average acoustic intensity; ISI, inter-stimulus interval; PRF, pulse repetition frequency; TBD, tone-burst duration.

Image-guided, reproducible placement of an acoustic focus to desired brain locations among awake animals was one of the important technical elements of the study. We developed a wearable, non-metallic (for MR-compatibility) FUS headgear for the sheep (exemplar photos of the headgear are shown in Figs. 1a,b). For repeated positioning of the frame across multiple FUS sessions, each of three support legs contained a 2-mm diameter groove that was aligned with tattoo markings made on the scalp (Figs. 1c,d). The tattoo markings, which were well-identifiable throughout the study period, served as fixed reference coordinates for the placement of the headgear. Once a ‘custom-fit’ headgear was made for one sheep, it was used only for that specific sheep throughout the experiments.

**Figure 2:**
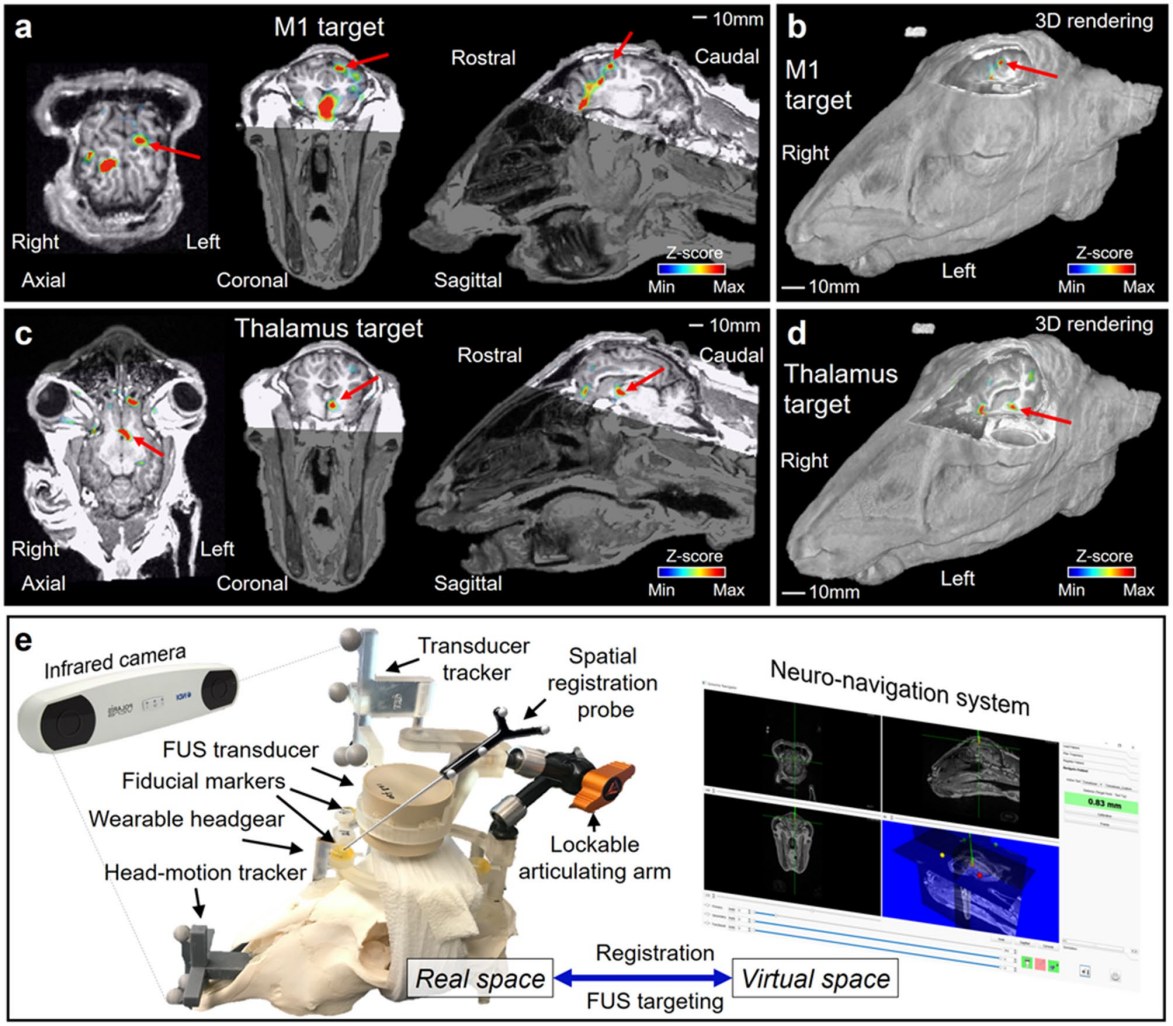
Schematics of the image-based sonication guidance and targeting. (**a–d**) Exemplary functional activation maps (uncorrected *P* < 0.05, *z*-score > 1.64) overlaid on the 3D anatomical neuroimage data, which show activation at the locations of (**a**, **b**) the M1 and (**c, d**) the thalamus denoted by red arrows. (**e**) An illustration of the neuro-navigation procedure using a sheep skull model. The figures (**a-d**) were created using Amira software (version 5.3.3, https://xtras.amira-avizo.com/).

Sonication to the M1 and the thalamus of the left hemisphere was guided by an in-house neuro-navigation system^13,21^ based on the acquisition of anatomical and functional MRI (fMRI) information from each sheep. fMRI-based identification of motor areas using a voluntary motor task is challenging in anesthetized animals. Therefore, tactile stimulation of the right hind limb muscle, which activates the corresponding motor area^11,22–24^, was adopted in fMRI to localize the functional motor area of the brain in the precentral gyrus and the thalamus, as supported by well-defined ovine functional neuroanatomy^25^. A functional activation map was co-registered to the volumetric anatomical MR image using the normalized mutual information^26^ (Fig. 2a–d). The real space coordinates of four MR fiducial markers were registered to those of the virtual MR space using an infrared motion-tracking system (Polaris Vicra, Northern Digital Inc., Waterloo, ON, Canada) (Fig. 2e), which was followed by alignment of acoustic focus to the target coordinates by maneuvering the transducer. The spatial accuracy of co-registration was evaluated by the fiducial registration error (FRE)^27^ whereby the FRE was 0.6 ± 0.1 mm (mean ± s.d.) through a total of 120 sessions across animals.

We evaluated the bimodal neuromodulatory effects of ultrasound to the left M1 and thalamus across three experimental conditions (on-/off-target FUS, and without sonication – ‘no-FUS’) across multiple sessions (120 FUS sessions with the time gap of 3.1 ± 2.7 days, mean ± s.d.). The order of the experimental conditions was randomized and balanced across the animals. Excitatory sonication was delivered at intervals of 5 s while the sheep stayed still. The pulsing scheme and parameters that lead to suppressive effects are substantially different from those typically used in conventional brain stimulation techniques such as in paired-pulse transcranial magnetic stimulation (pp-TMS) protocols^28,29^, and typically require much longer sonication duration, on the orders of tens of seconds and even minutes^1,2,7,10,30^. Therefore, suppressive effects are not readily achieved by delivering short bursts of sonication in a time-locked fashion. Consequently, suppressive sonication was delivered for a duration of one minute when the sheep walked/strolled on a treadmill at a constant gait speed of 0.5–0.8 mph, adjusted for each animal.

Telemetry EMG data were acquired from gastrocnemii of both hind limbs. A single-element FUS transducer (the focal length was 30 mm from the exit plane) was operated at a fundamental frequency of 250 kHz, whereby its focal dimension was 13 mm in length and 3 mm in diameter, with estimated ellipsoidal volume of 0.06 mL (measured at 90%-maximum of the intensity; Fig. 1g). The 90%-maximum of the intensity profile was based on the previous studies that showed the neuromodulatory area was far more localized than the spatial profile defined by full-width at half-maximum (FWHM)^31,32^. These studies detected the area showing elevated glucose metabolism in response to FUS stimulation in rats through positron emission tomography, revealing its profile being approximated at full-width at 90%-maximum of the acoustic intensity. The focal size defined at FWHM of intensity was 56 mm in length and 10 mm in diameter, with an estimated volume of 2.93 mL. The in situ spatial-peak pulse-average intensity (I_sppa_) was estimated using an intensity transmission level of 25.6% derived from skull transmission at 250 kHz^10,13^. We utilized optimal sonication parameters (Fig. 1f for illustration) that were identified from our previous study in anesthetized sheep^10^. In detail, for the excitatory sonication, 200-ms sonication duration, 0.5-ms tone-burst duration (TBD), and 70% duty cycle (DC) were used. For the suppressive sonication, 1-min sonication was given using 0.5-ms TBD and 5% DC. Before initiating experiments on each animal, the test sonication (~ 5 excitatory sonications or ~ 10 s-long suppressive sonication) was delivered, starting from I_sppa_ of 20.5 W/cm^2^ for excitation or 13.7 W/cm^2^ for suppression (all expressed in situ value). If the animal showed any signs of disturbance (such as head shaking or sudden movement), we reduced the intensity until the sign was not observed. As a result, excitatory sonication was given at I_sppa_ of 5.2 W/cm^2^ (*N* = 6 sheep; corresponding to 3.6 W/cm^2^ spatial-peak temporal-average intensity, I_spta_) or 20.5 W/cm^2^ (*N* = 4; 14.4 W/cm^2^ I_spta_). Suppressive sonication was given at I_sppa_ of 1.5 (*N* = 1; corresponding to 0.1 W/cm^2^ I_spta_), 4.7 (*N* = 2; 0.2 W/cm^2^ I_spta_), 5.3 (*N* = 6; 0.3 W/cm^2^ I_spta_) or 13.7 W/cm^2^ (*N* = 1; 0.7 W/cm^2^ I_spta_). In the off-target FUS condition, we delivered sonication to the parietal brain regions ~ 10-mm caudal and ~ 5-mm lateral to the target M1 area, in which fMRI showed no activation. For the condition without any sonication, the entire experimental procedure was repeated in the absence of sonication.

### Analysis of EMG elicited by excitatory FUS sonication

In each of the excitatory sessions, sheep were subjected to multiple runs of experiments (25.8 ± 3.3 runs, mean ± s.d., *N* = 10 sheep) where 10 excitatory FUS sonication events (defined as ‘trials’) were given in each run with 5-s inter-stimulus interval. We did not observe visible movement of the hind limbs elicited by excitatory FUS stimulation from any of the animals.

**Figure 3:**
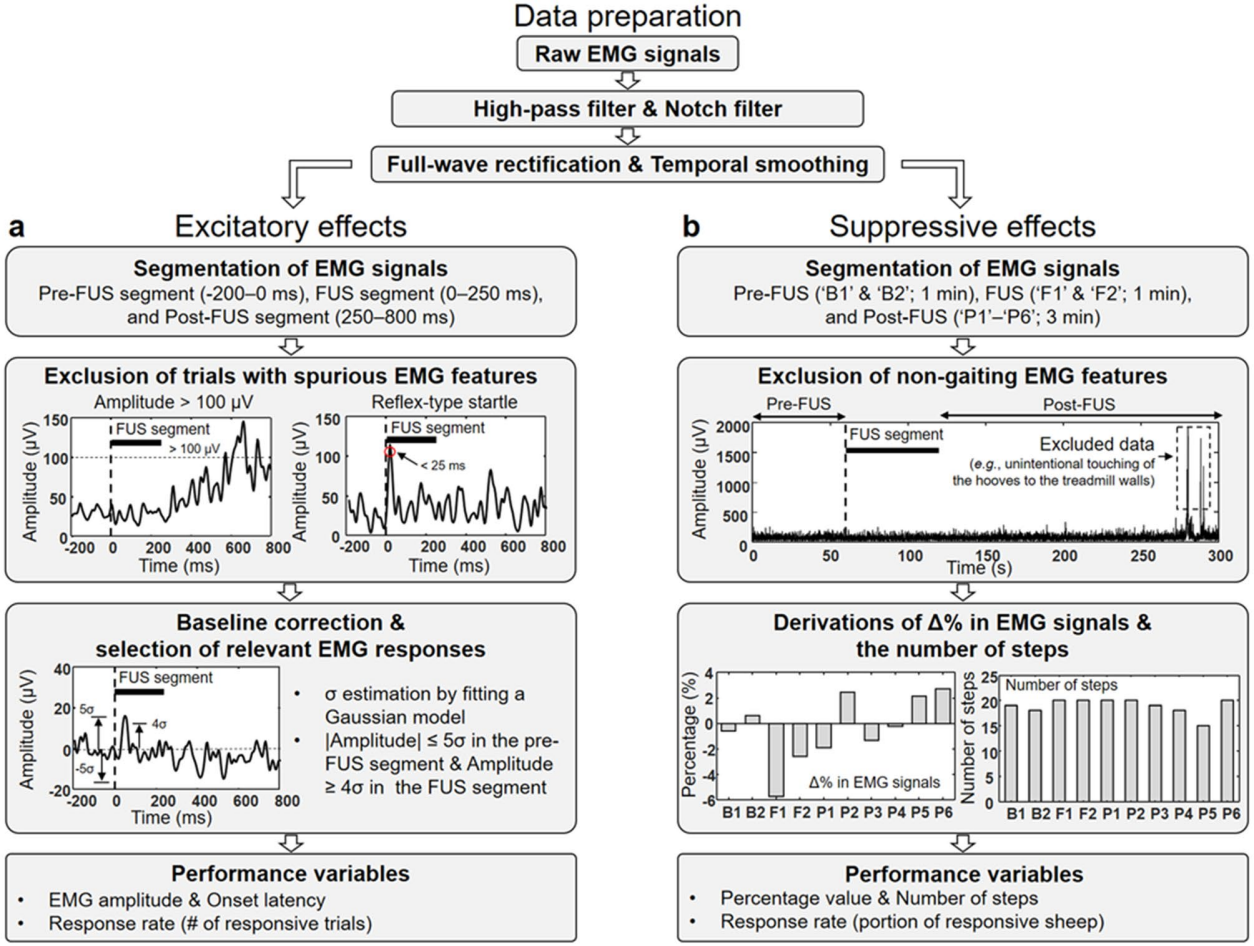
A flow chart of EMG processing and analysis. After preparing the raw EMG data with high-pass/notch filtering, full-wave rectification, and temporal smoothing (noted as ‘Data preparation’), the EMG data were subjected to two analysis pathways to derive the performance variables to evaluate (**a**) excitatory and (**b**) suppressive effects. σ is the standard deviation of the fitted Gaussian distribution (Supplementary Fig. S1). Δ%, percentage difference; EMG, electromyography; FUS, focused ultrasound.

EMG data analysis from awake large animals without physical constraints, especially in the context of FUS-mediated neuromodulation, casts a unique set of challenges. As there is no standardized method for analyzing such data, we adopted several well-characterized methods (filtering, rectification, and temporal smoothing) to prepare the data and added selection criteria to exclude motion-related features irrelevant to sonication (as described in Fig. 3a for illustration). Then, the prepared EMG signals were time-locked between -200 ms to 800 ms with respect to the onset of each sonication trial. Subsequently, time-locked periods were further divided into three segments—pre-FUS (-200–0 ms), FUS (0–250 ms) and post-FUS (250–800 ms). The rationale for defining the duration of these time segments was based on our previous study on anesthetized sheep, which demonstrated EMG responses observed in the range of 25–250 ms upon sonication onset^10^. Considering the conduction velocities of the peroneal and tibial nerves (~ 100 m/s, which yields ~ 15–20 ms latency for signal conduction from the brain to hind limbs)^33^, we anticipated that most of the efferent EMG signals associated with the stimulation would be captured in the range of 25–250 ms. The EMG data from each FUS trial were screened for the presence of (1) signal saturation (*i.e.*, absolute value ≥ 100 µV), (2) a single peak with short latencies (< 25 ms, which is related to the possible presence of reflex-type startle), and (3) multiple peaks or synchronous EMG signals appeared from both hind limbs within the FUS segment, which were indicative of spurious motion-related artifact that are related to simple postural change.

The numbers of the total EMG data after the screening were 223.9 ± 35.3, 229.1 ± 31.2, 219.2 ± 39.2 and, 224.8 ± 21.5 (mean ± s.d., *N* = 10) for the on-target M1, thalamus, off-target FUS, and no-FUS conditions, respectively. The numbers of selected data were not statistically different across experimental conditions (one-way analysis of variance [ANOVA], *F*(3,36) = 0.2, *P* = 0.92). Then, a histogram of EMG amplitudes across the trials of the pre-FUS segment was plotted. A Gaussian distribution was fitted to the histogram, with the mean value set as the mode value of the EMG amplitude distribution, whereby its standard deviation (σ) was used to derive the degree of baseline EMG signal fluctuation during resting-state. The fitting was needed since the EMG amplitudes obtained from pre-FUS/resting segments showed positively-skewed distributions in the presence of resting-state muscle activity (Supplementary Fig. S1).

Subsequently, the entire EMG time series from each trial were baseline-corrected with respect to the mean obtained from the pre-FUS segment, and the corrected EMGs that are associated with the sonication (not necessarily being synchronized with the sonication onset) were selected using the following inclusion criteria: (1) |amplitude|≤ 5σ during the pre-FUS segment and (2) amplitude ≥ 4σ in the FUS segment. Then, the selected data was averaged per each animal to represent responsive EMG feature. The numbers of EMG data sets per animal that were identified to be associated with sonication were 16.4 ± 2.5, 15.0 ± 2.3, and 16.6 ± 3.2 (mean ± s.d., *N* = 10) for M1 on-target, thalamus on-target, and off-target conditions, respectively, without bias in selection (one-way ANOVA, *F*(2,27) = 1.1, *P* = 0.36).

### Comparison of EMG amplitudes between the hind limbs

**Figure 4:**
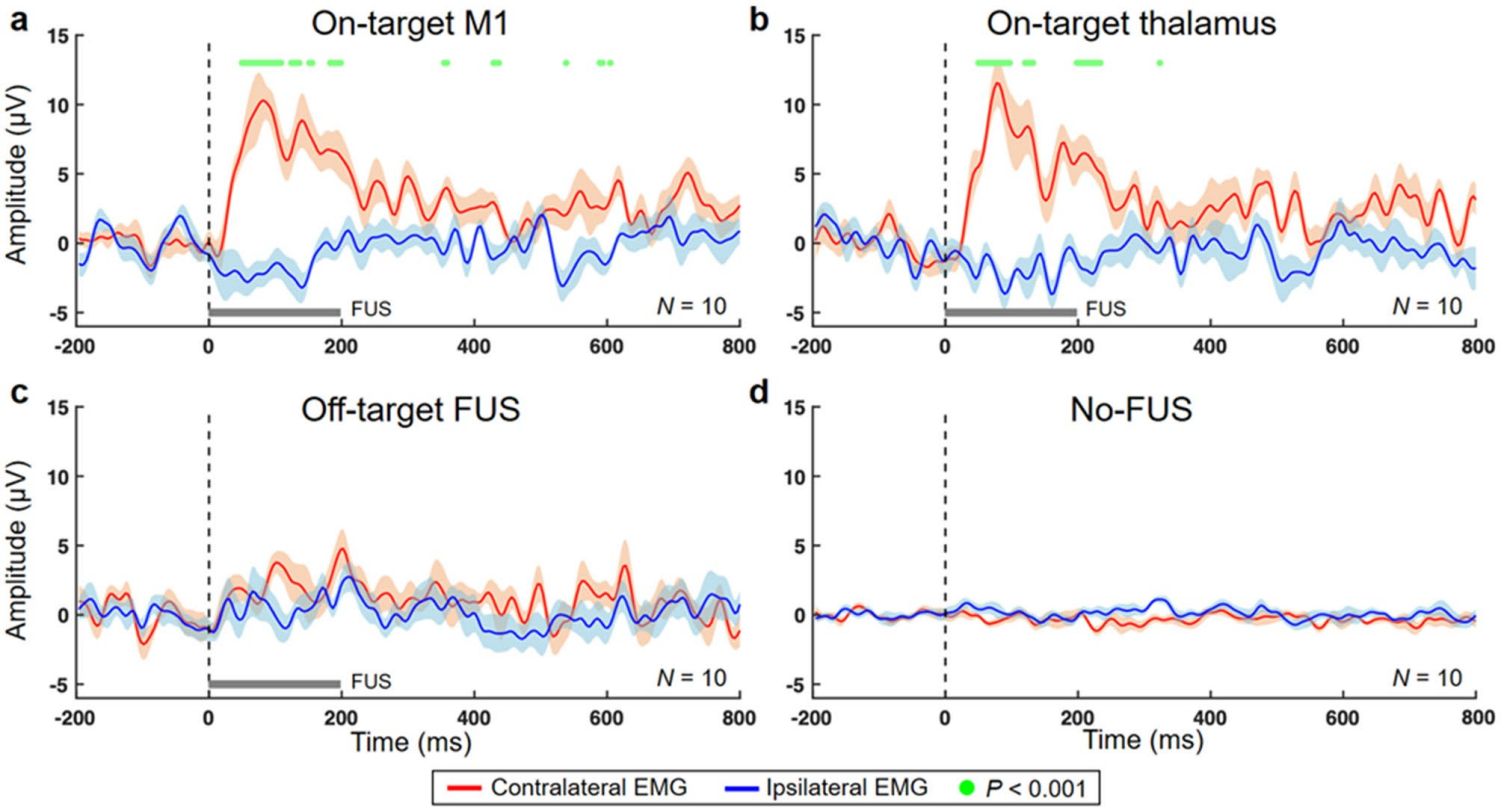
Comparison of time-locked EMG amplitudes between the hind limbs. The group-averaged EMG amplitudes measured from the hind limbs contralateral (red line) and ipsilateral (blue line) to 200-ms sonication (gray bar) were plotted (line: average across *N* = 10 sheep, shaded areas: standard errors) from stimulation of (**a**) the M1, (**b**) the thalamus, and (**c**) off-target area. (**d**) The same plot representing the no-FUS condition is shown. The green dots indicate significant differences (paired *t-*test, *P* < 0.001) in the EMG amplitudes between the hind legs.

Group-averaged amplitudes of the EMG signals obtained from the hind limbs across the experimental conditions are shown in Fig. 4. In the M1 stimulation, the EMG amplitudes measured from the right hind limb contralateral to sonication were significantly greater than those measured from the ipsilateral (left) hind limb mainly during 50.0–198.4 ms after the FUS onset (paired *t*-test, *P* < 0.001 presented by green dots in Fig. 4a). Likewise, sonication of the thalamus significantly increased the contralateral EMG amplitudes compared to the ipsilateral EMG amplitudes mainly within 50.0–132.0 ms after the FUS onset (paired *t*-test, *P* < 0.001, Fig. 4b). In the cases of the off-target and no-FUS conditions, there were no statistical differences in the EMG amplitude between the hind limbs (Figs. 4c,d).

### Comparison of EMG amplitudes among experimental conditions

We compared the group-averaged amplitudes of the EMG signals from each hind limb among experimental conditions (on-target, off-target and no-FUS), and showed that the on-target sonication of the motor circuits selectively increased the EMG amplitudes from the hind limb contralateral to the FUS while comparisons between on-/off-target and no-FUS conditions yielded no statistical differences from the ipsilateral (left) hind limb (Supplementary Results and Fig. S2).

### Latency distribution of EMG elicited by excitatory FUS sonication and derivation of response rate

**Figure 5:**
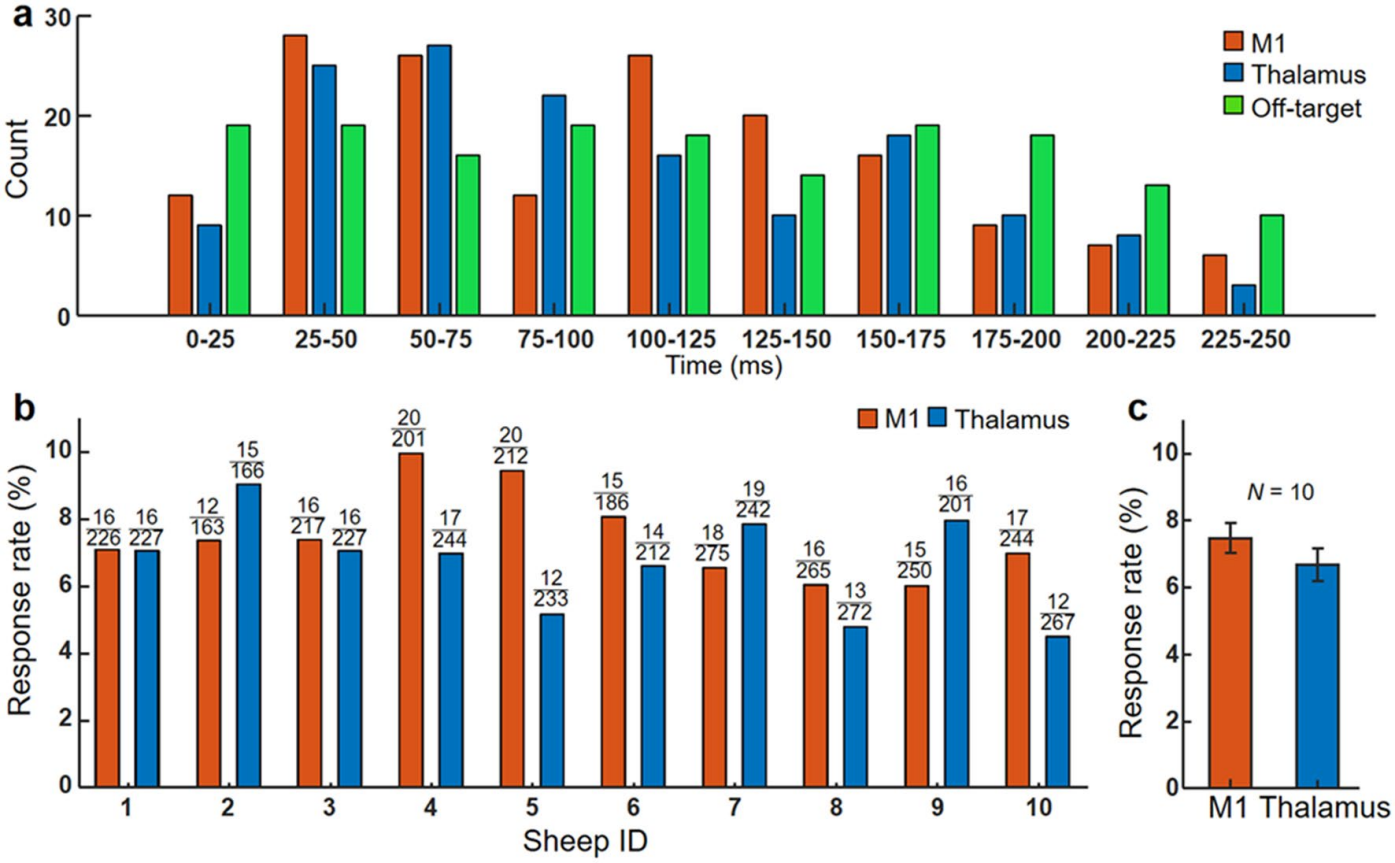
Latency distribution and response rate of EMG elicited by excitatory FUS sonication. (**a**) A histogram of onset latencies of selected EMG signals after the FUS onset across all animals. The signals were obtained from the hind limb contralateral to sonication. (**b**) Response rates in excitatory sonication to the M1 (red bars) and thalamus (blue bars) from each animal and (**c**) their average across all sheep (*N* = 10). The error bars indicate standard errors. The numbers above each data bar represent response counts (numerator) and the total number of trials (denominator).

Analysis of EMG from the right hind limb elicited by excitatory on-target FUS revealed elevated EMG amplitude compared to the off-target condition. We constructed a frequency histogram of onset latency of selected EMG signals that are associated with sonication (both on- and off-target sonications) for every 25-ms latency bin up to 250 ms (10 bins) across all animals (Fig. 5a). The EMG onset latency was defined as the time when EMG exceeded the resting-state σ after the sonication onset. The onset latency values in the range from 25–50 and 50–75 ms were most frequently observed from the M1 and thalamic stimulations, respectively. The averaged latencies from the EMG signal were 102.0 ± 20.7 ms for the M1 and 101.0 ± 17.4 ms for the thalamus (mean ± s.d., *N* = 10). Off-target sonication, on the other hand, yielded more uniform latency distribution (green bar in Fig. 5a), suggesting selected EMG peaks are not synchronized with sonication.

Based on the time-synchrony with sonication, the selected trials and corresponding EMG associated with on-target conditions were identified as being responsive to FUS and were used to derive the successful excitatory response rate per animal (with respect to the number of trials without the presence of signal saturation). Sheep-specific response rate shows a degree of variability (Fig. 5b), with averaged response rates of 7.5 ± 1.4% from the stimulation of the M1 (ranging from 5.7–10.0%) and 6.7 ± 1.5% from the stimulation of the thalamus (4.5–9.0%) (Fig. 5c). The response rates were not statistically different between the sonications of the M1 and thalamus (two-tailed *t*-test, *P* = 0.26, *N* = 10).

### Analysis of suppressive sonication

To evaluate the effects from suppressive FUS, the sheep voluntarily walked on a treadmill at a constant gait speed under the monitoring of EMG for a total of 5 min, which consisted of three segments (1 min pre-FUS, 1 min with FUS and 3 min post-FUS). The animal’s gait was also video recorded using a camera placed on the center of the treadmill console. After data preparation (*i.e.*, filtering, rectification, and temporal smoothing, illustrated in Fig. 3), the EMG signals were divided into 30-s segments—pre-FUS (‘B1’ and ‘B2’), FUS (‘F1’ and ‘F2’), and six post-FUS segments (‘P1’ through ‘P6’). The data processing scheme is illustrated in Fig. 3b. The video data were cross-referenced to exclude EMG signals contaminated by non-gaiting behaviors, such as an unintentional touching of the hooves to the treadmill walls and distracted responses to external sound noises (*e.g.*, another sheep bleating) whereby five time segments were excluded out of a total of 400 (*i.e.*, 10 sheep × 4 experimental conditions × 10 time segments). The durations of excluded EMG signals from analysis were 1.9 ± 3.3 s (B1), 2.0 ± 3.5 s (B2), 2.8 ± 6.7 s (F1), 2.5 ± 7.4 s (F2), 1.7 ± 4.8 s (P1), 1.7 ± 4.7 s (P2), 3.8 ± 8.2 s (P3), 2.7 ± 6.5 s (P4), 2.7 ± 5.6 s (P5), and 4.7 ± 7.7 s (P6) (mean ± s.d., *N* = 10). There were no statistical differences in excluded EMG signals across time segments (one-way ANOVA, *F*(9, 81) = 0.4, *P* = 0.93).

Since each animal received suppressive sonication for each condition, the number of animals that exhibited suppressive responses were measured. To define a successful response, we first calculated a standard deviation of the percentage values obtained from each animal’s pre-FUS segments (*i.e.*, ‘B1’ and ‘B2’). Then, success was determined if the percentage value obtained from a sonication segment (either ‘F1’ or ‘F2’) decreased more than 1.73 times of the standard deviation (corresponding to *P* < 0.05, one-tailed). All 10 sheep showed suppressive responses to sonication of either M1 or thalamus. Seven sheep showed responsive suppression from sonicating the M1 whereas eight responded to thalamic suppression (Individual data shown in Supplementary Table S1).

There was no significant difference in the number of steps taken across the experimental conditions (Supplementary Fig. S3; one-way ANOVA, the right hind limb, *F*(3,36) = 2.0, *P* = 0.13; the left hind limb, *F*(3,36) = 1.2, *P* = 0.33). In addition, none of the experimental conditions showed significant time-dependent (across ‘B1’– ‘P6’, one-way repeated measures ANOVA, *F*(9,81) = 0.7–2.0, *P* > 0.05) or limb-specific trends (paired *t*-test, *P* > 0.05, *N* = 10) in the number of steps.

### Analysis of percentage difference in EMG amplitudes

#### Within-condition comparison among time segments and between hind limbs

**Figure 6:**
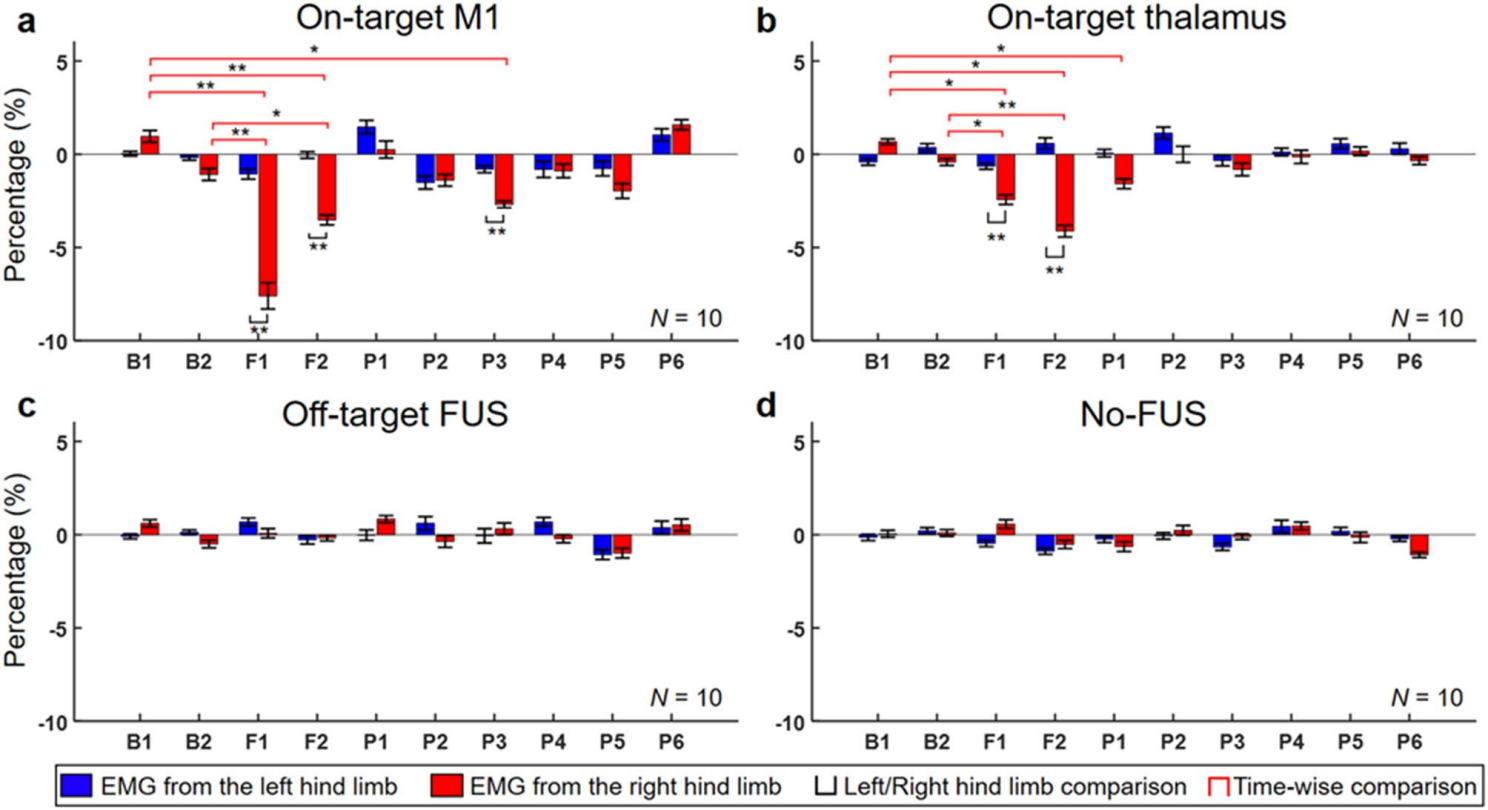
Within-condition comparisons among time segments and between hind limbs in suppressive sonication sessions. Percentage differences in the EMG amplitudes before (B1 and B2), during (F1 and F2), and after (P1–P6) suppressive sonication to (**a**) the M1, (**b**) the thalamus, (**c**) the off-target area, and (**d**) data from no-FUS condition (*N* = 10 sheep). A one-way repeated measures ANOVA (*P* ≤ 0.001 for the right hind limb with on-target M1/thalamus conditions) with Tukey–Kramer *post-hoc* analysis for multiple comparisons was conducted to analyze the time-dependent differences in the amplitude obtained from each hind limb (left, blue bar; right, red bar). No statistical differences were shown from the right hind limb during off-target and no-FUS conditions, and from the left hind limb during all the experimental conditions. To compare the difference in the amplitudes between the hind limbs, paired *t*-test was conducted. The bars and error bars indicate average values and standard errors, respectively. ^*^P < 0.05, ^**^P < 0.01.

The percentage difference in EMG signal activity for each time segment (denoted as ‘Δ%’ in Fig. 3b) was calculated as the ratio of the mean signal value with respect to the mean value of the two baseline segments (B1 and B2). Suppressive FUS of the M1 yielded significant differences in the EMG amplitudes across time segments (one-way repeated measures ANOVA, *F*(9,81) = 4.8, *P* < 0.001; Fig. 6a). Subsequent Tukey–Kramer *post-hoc* analysis revealed significantly decreased (*P* < 0.05 presented by red brackets) EMG amplitude obtained from the right hind limb (contralateral to sonication) in the F1 (− 7.6 ± 7.1%, mean ± s.d., *N* = 10), F2 (− 3.5 ± 2.6%), and P3 (− 2.7 ± 1.8%), compared to the one from the B1 segment (1.0 ± 3.1%). In addition, a significant reduction in EMG amplitudes (*P* < 0.05) was also found in the F1 and F2 when compared to the amplitude obtained in the B2 (− 1.1 ± 3.2%). These differences were not observed across P4–P6 time segments, which indicate that the EMG amplitude returned to the baseline level around 90-s post-sonication. When the EMG amplitudes were compared between the hind limbs in each time segment, the amplitudes obtained from the right hind limb were significantly reduced (*P* < 0.01 presented by black brackets) compared to those from the left hind limb during the F1 (− 1.1 ± 2.7%), F2 (0.0 ± 1.8%) and P3 segments (− 0.8 ± 1.9%). However, the differences were not observed in other time segments.

Suppressive FUS of the thalamus generated a similar trend to the sonication of the M1, showing significant differences in the EMG activity among time segments (one-way repeated measures ANOVA, *F*(9,81) = 3.6, *P* = 0.001; Fig. 6b). *Post-hoc* analysis showed that the EMG amplitudes obtained from the right hind limb were significantly decreased (*P* < 0.05) during the FUS (− 2.4 ± 2.5% in the F1 and − 4.1 ± 3.2% in the F2) and P1 segments (− 1.6 ± 2.4%), compared to those from the B1 (0.7 ± 1.5%). A significant decrease (*P* < 0.05) in the EMG signal during the FUS segments (F1 and F2) were also found compared to the amplitude obtained from the B2 (− 0.4 ± 1.8%). From the comparison between the hind limbs, the EMG amplitude obtained from the right hind limb (contralateral to stimulation) was significantly reduced (*P* < 0.05) compared to one from the left during the FUS segment (− 0.6 ± 1.6% in the F1 and 0.6 ± 3.0% in the F2; Fig. 6b). None of the off-target or no-FUS conditions showed any statistical differences among time segments and between hind limbs (Figs. 6c,d). The results suggest the selective and temporary reduction of EMG amplitudes contralateral to sonication.

### Hind-limb specific comparisons across the experimental conditions in each time segment

We performed hind-limb-specific comparisons of the EMG amplitude across the experimental conditions and observed that suppressive sonication selectively decreased EMG amplitudes from the hind limb contralateral to sonication of the motor circuits (Supplementary Results and Fig. S4). Also, the suppressive effect from sonication of the M1 was shown during the P3 segment compared to the amplitude from the off-target condition.

### Effect of acoustic intensity on response rates and evaluation of thermal effects

The effects of different acoustic intensities on the neuromodulatory outcomes were evaluated in terms of the response rate and maximum EMG amplitude during FUS segment from excitatory sonication, and percentage reduction in EMG amplitude (during F1 segment) from suppressive sonication (Supplementary Fig. S5). Although a weak correlation between acoustic intensity and response rates was found from excitatory sonication (*P* = 0.1), no significant correlation was identified across the measures.

Although a higher intensity has been used without raising tissue temperature in sheep^10^, the ultrasonic stimulation was administered on the M1 area which is close to the skull, and the potential risks of skull heating were evaluated. Temperature change at the sonicated M1 and adjacent skull was estimated by sequentially solving the Khokhlov-Zabolotskaya-Kuznetsov (KZK) and bio-heat transfer Eqs. ^34^, following the method described in Supplementary Methods (see “*Evaluation of thermal effects*”). The estimation was performed using the maximum in situ acoustic intensity for each of excitatory (20.5 W/cm^2^ I_sppa_) and suppressive sonication (13.7 W/cm^2^ I_sppa_), which revealed negligible thermal rise in the brain tissue as well as in the skull (≤ 0.0003 °C; Supplementary Fig. S6).

### Post-sonication behavior monitoring and histological assessment

**Figure 7:**
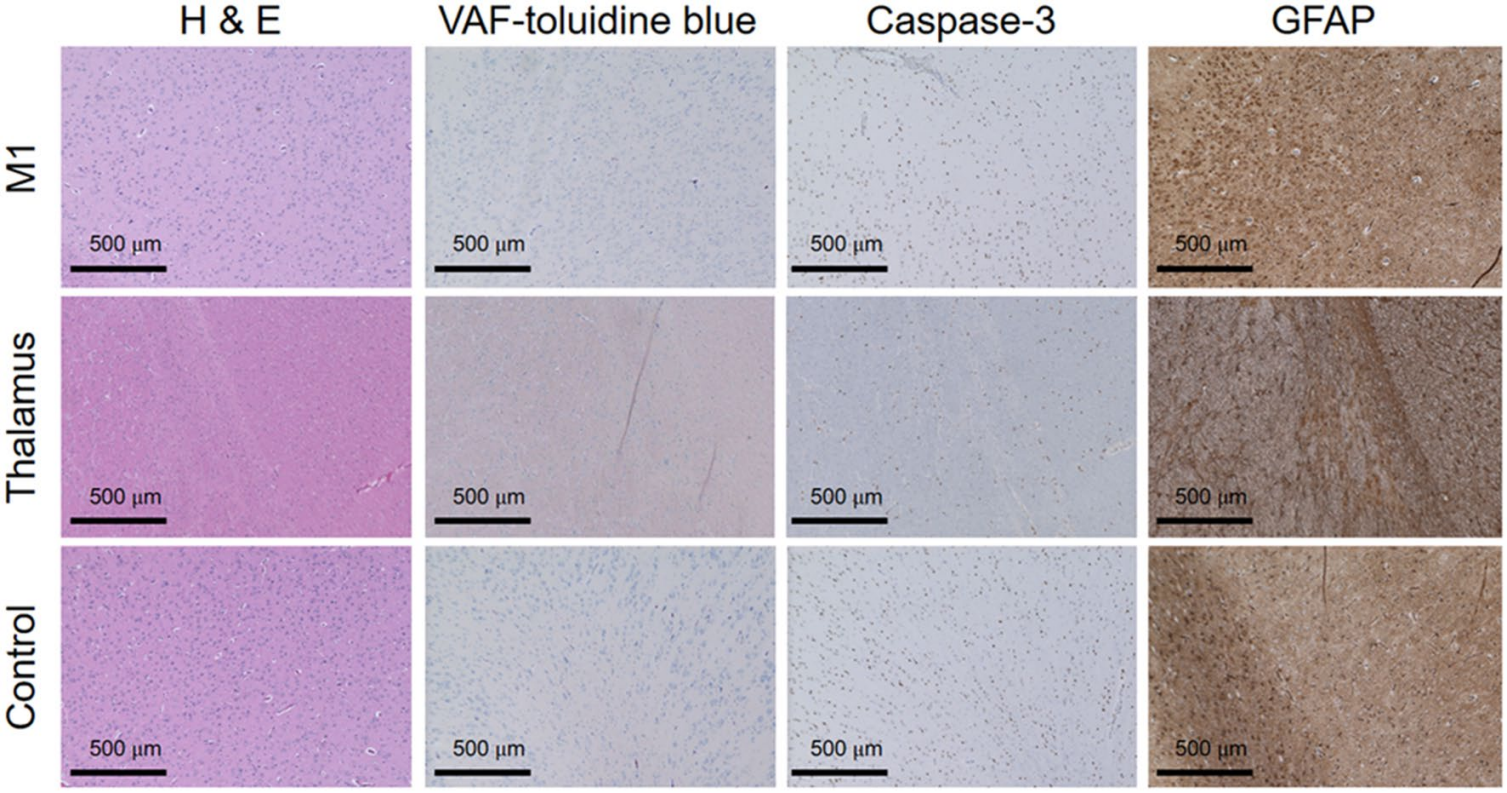
Exemplary histology analysis results of brain tissues. The microscopic images (40 × magnification) of the M1, thalamus, and control sites are displayed across staining methods, H&E, VAF-toluidine blue, Caspase-3, GFAP. No evidence of tissue damage due to FUS sonication is observed.

All animals showed normal behavior with no loss of appetite and weight throughout the study period. We did not find any sign of brain tissue damage from histological analysis using hematoxylin and eosin (H&E), vanadium acid fuchsin (VAF)-toluidine blue staining and immunohistochemistry (IHC) of glial fibrillary acidic protein (GFAP) and caspase-3 across different time intervals after the last sonication session. Figure 7 shows representative microscopic images of the histological slides.

## Discussion

### Wearable FUS headgear and training sessions

We developed FUS headgear that was worn by awake sheep to administer FUS to the M1 and its thalamic projection in a spatially reproducible fashion, as guided by individual functional neuroanatomy. Instead of the surgical approach to implant a pedestal to attach a FUS transducer, despite its success in a rodent study^20^, the use of the sheep-specific headgear positioned over the three reference points tattooed on the scalp not only allowed for reproducible placement of the FUS transducer, but also significantly promoted the well-being of the animals. We believe that the effect of head growth on the positioning of the headgear was minimal as the headgear was well-aligned to the tattooed markings throughout the study and the FRE remained small (0.6 ± 0.1 mm, mean ± s.d.) through a total of 120 sessions across animals. We also emphasize the importance of acclimation (to the vest, the mock headgear, and temporary bandaging) as well as recurring training sessions to keep animals compliant with behavioral requirements for the duration of the study.

### Rationale behind experimental design

In the present study, excitatory sonication was delivered as a short (200 ms-long) burst in a time-locked fashion to stationary sheep whereas 1-min long suppressive sonication was administered to animals walking on a treadmill. Therefore, a transposition of the experimental design might have been sought after, for example, excitatory sonication administered to walking animals at a specific gait cycle. It is reasonable to hypothesize that the excitatory sonication may increase EMG tones during treadmill walking. However, the use of a higher duty cycle (*e.g.*, 70% for excitation) than the one used for suppression (5%) inherently increases the risk for heat generation in the brain and the adjacent skull due to higher level of acoustic energy deposition. Apart from the unknown safety of implementing such a design, it also requires identification and sonication of the brain motor area relevant to the gait-specific muscle groups. Another alternative experimental design is to deliver short burst of suppressive sonication in a time-locked fashion; however, unlike excitatory acoustic stimulation, suppressive effects are not likely to take place within a gait cycle, which is typically less than a second^1,2,7,10,30^. Furthermore, the design would require technical advancement enabling automated gait analysis and synchronized FUS delivery. These designs, although extremely challenging to implement, are theoretically feasible and anticipated to provide further information on mechanism behind the acoustic neuromodulation.

### Examination of excitatory effects on awake sheep: EMG features and response rate

On-target administration of FUS to the M1 or the thalamus selectively elevated EMG amplitude from the hind limb contralateral to stimulation while the off-target stimulation did not (Fig. 4 and Supplementary Fig. S2). This finding suggests that FUS stimulated the motor circuit in a spatially selective fashion and generated efferent output detected by the EMG in awake sheep. Several EMG peaks were detected during the stimulation of the non-motor parietal area (off-target condition), an area that is comparable to the somatosensory association area of the human brain. However, their latencies were rather evenly distributed across the observed time window upon stimulation (Fig. 5a), suggesting the presence of spontaneous muscle activity from either/both hind limbs while they stand still or change posture.

On the other hand, the latency distribution from the hind limb contralateral to sonication showed a degree of synchrony with respect to the sonication. Stimulation of the M1 and thalamus yielded elevated EMG peaks in the range of 42 − 109 ms (after the sonication onset) compared to the off-target/no-FUS conditions. The range was slightly shorter than the results from the previous experiment on the anesthetized sheep^10^ (*i.e.*, 100–190 ms). Further examination of latency distributions of EMG onset with respect to the sonication onset showed most occurrences in a latency segment of 25 − 75 ms, for both M1 and thalamic stimulation (Fig. 5a). The results indicate that a larger portion of responses occurred slightly earlier in awake sheep compared to those obtained from anesthetized sheep in terms of onset latency distribution^10^. These results share similarities with a rodent study that demonstrated a shortened response latency in the awake state^20^ and may indicate that the absence of anesthesia yields faster efferent responses to excitatory FUS stimulation.

The averaged EMG response rates from the right hind limb elicited by the M1 and thalamic stimulations among awake sheep were 7.5 ± 1.4% and 6.7 ± 1.5%, respectively (Fig. 5c). The response rates were comparable to or even higher than the values measured from acoustic brain stimulation to elicit hind leg response in mice (ranging from 1.8–8.2%)^19^, but were slightly lower than 12.4% (from M1 stimulation) and 6.9% (from thalamic stimulation) in anesthetized sheep using a similar pulsing scheme^10^. The response rates from the present study were also lower than those typically observed in TMS^35,36^. Furthermore, the stimulation did not elicit any visible movement of the hind limb, while various physical movements have been observed in acoustic stimulation of rats or mice (*e.g.*, the whiskers, limbs, or tail)^5,18,20,37^. Although responses might have been underestimated during the data selection process in the present study (*e.g.*, exclusion of multiple peaks or synchronous EMG signals appeared from both hind limbs within the FUS segment), we speculate that the lower EMG responses rates, along with the absence of visible movements, may be attributed to anatomy-specific variations in number and types of efferent motor output neurons^38^, combined with an insufficient number of recruited motor units at peripheries. Despite the use of neuroimaging guidance with excellent spatial accuracy and fMRI-based localization of animals’ functional motor circuits, distortion of the acoustic propagation through the skull confounded by the elongated focal shape^10,13^, might have also contributed to misalignment of acoustic focus to the M1 and thalamus, including being overlapped with adjacent somatosensory area, creating a suboptimal condition for stimulation.

Reproducible positioning of the acoustic focus is also affected by the skull, which may distort acoustic wave propagation. We applied the FUS wave direction as perpendicular to the skull surface as possible to minimize the contribution from the skull^13^. However, non-invasive validation of spatial precision in sonication, obtained by utilizing MR thermometry after moderate tissue heating^1,39^ or MR-acoustic radiation force imaging (ARFI)^40^ as examples, is desired. Temperature-related changes in neuronal excitability in MR-thermometry and lack of detection sensitivity toward the use of low-intensity in MR-ARFI are needed to be addressed prior to application. A numerical simulation to estimate the location and pressure level *in situ*^13,41^, based on the acquisition of geometry and composition of cranial structure using X-ray computed tomography (CT), may be adopted for accurate targeting. Phased-array FUS transducers that are capable of correcting for phase aberration due to skull^42,43^ may be considered in the future; however, their (relatively large) size would impose technical challenges in implementing among awake animals. Taken together, although the present study enabled acoustic brain stimulation in awake ovine model which provide translational information to future human studies (desired to be performed while subjects/patients are awake), further methodological challenges remain to be addressed, especially in reducing the spurious EMG signals and improving the accuracy of sonication.

Regarding the variability of response rate across the awake sheep (Fig. 5b), we observed smaller variability in responses across the animals compared to those previously measured under anesthesia (12.4 ± 9.2% across 8 sheep)^10^. The reduced level of response variability in the awake state agrees well with the previous rodent investigation^20^, and suggests that anesthesia may indeed confound the responses to brain stimulation, with greater inter-animal variability.

### Examinations on suppressive effect on awake sheep – Temporary reduction of EMG amplitudes

While sheep walked on the treadmill, suppressive FUS administered to the M1 or thalamic area reduced the amplitudes of EMG from the hind limb for the periods of F1, F2, and P3 for the M1, and F1, F2, and P1 for the thalamus, compared to pre-sonication segments (Figs. 6a and b), specifically to the contralateral limb (Supplementary Fig. S4a). The percentage amplitude reduction in EMG bears similarities with our previous study on modulation of somatosensory evoked EEG potential (SSEP, induced by passive electrical stimulation) by FUS on the primary sensory area (S1) and corresponding thalamic projection among anesthetized sheep^10^. The attenuation of SSEP amplitudes by the suppressive FUS to the S1 was also reported in humans^30^. In small animal studies, similar suppressive effects of FUS have been demonstrated in rats and rabbits (visual area)^1,4^ as well as sonicating the lateral geniculate nucleus in cats^44^.

The reductions in EMG amplitude, however, did not alter behavioral gait patterns (*e.g.*, number of steps taken, Supplementary Fig. S3), which may indicate that the observed level of EMG reduction may not reach the level that affects gaiting (*i.e.*, the number of steps) of animals who are required to walk on the treadmill at a fixed speed. Further studies would be warranted to correlate electrophysiological recordings with behavioral outcomes during the delivery of suppressive FUS. The use of faster (*i.e.*, more challenging to animals) gait speed along with motion characterization would be beneficial for kinematic analysis (*e.g.*, gait cycles, including stride, stance, or swing) to provide more detailed information on behavioral changes associated with the awake experimental setting^45^. Alternatively, concurrent wireless EEG measurements may also help verify the responses elicited by FUS stimulations from freely moving awake sheep.

The suppressive effects on EMG outlasted the 1-min duration of sonication and were reversible as the decreased EMG amplitudes were recovered to the pre-FUS baseline level in P4 segment (90 − 120 s) after the suppression of M1 and in P2 segment (30 − 60 s) after the thalamic suppression (Figs. 6a,b, and Supplementary Fig. S4). These results are also in agreement with our previous work on anesthetized sheep showing that SSEP magnitude reduced by 2-min suppressive FUS to the S1 was restored within 5 min after sonication^10^. In a swine model (under anesthesia), Dallapiazza and colleagues reported that 40-s pulsed FUS applied to the ventral posterolateral nucleus of the thalamus sustainably suppressed SSEP amplitude up to 5 min, then was later returned to the baseline level in 10 min^7^. Our results also support the effects of a 40-s FUS stimulation delivered to the supplementary motor area or frontal polar cortex of non-human primates on modulating their brain cortical activation and connectivity more than one hour after sonication^8^. Taken together, these results suggest that exposure to FUS may result in modulatory effects that far-outlast the sonication duration itself, which may be conducive to inducing neural plasticity. We previously reported that 10-min suppressive FUS to the S1 in anesthetized rats showed lasting effects yielding differential SSEP beyond 35 min after sonication^6^, whereby 30 min duration of altered neural state is known to induce initial long-term potentiation or long-term depression-like synaptic behavior^46,47^.

### Acoustic parameters and thermal effects

We found that the sonication parameters used in the present study, which were previously identified from anesthetized sheep, generated bimodal neuromodulatory effects in awake sheep. The sonication parameters were also comparable to the ones identified through our previous studies, for example, 50% DC, 0.5–1-ms TBD, and 300–500 ms sonication duration to achieve excitatory effects^11,37,48–50^ and 5% DC, 0.5-ms TBD, and 18–150 s sonication to induce suppressive effects^1,4,10^. Similar parameters have been shown to induce excitatory effects in mice (50% DC, 0.5-ms TBD, and 1 s sonication)^51^ and to suppress auditory evoked potentials in rats (3% DC, 100-ms TBD, and 52-s sonication duration)^52^.

We note that downward adjustment of the acoustic intensity was needed to conduct experiments in awake sheep. tFUS experiments in awake animals cast unique and unexpected challenges compared to anesthetized sheep, whereby the individual responses toward acoustic intensity varied substantially. For example, 3.6 W/cm^2^ I_spta_, much lower than the intensity used in the previous study (11.1 or 12.7 W/cm^2^ I_spta_), was used across six sheep due to signs of behavioral disturbance, while other four underwent the experiment without disturbance even at a slightly higher intensity of 14.4 W/cm^2^ I_spta_. In suppressive sonication, we used lower intensities (0.1–0.3 W/cm^2^ I_spta_ in 9 out of 10 sheep) compared to those used in the previous study in anesthetized sheep to avoid disturbance in treadmill walking. Direct correlation of specific sheep behavior to humans is difficult. For example, sheep generally do not want to be touched on their head (even gently), resulting in avoidance or head shaking behaviors. Therefore, the behavior we observed during sonication may not necessarily be due to the ‘unpleasant’ or ‘painful’ sensations, but instead, it may have simply stemmed from any circumstances not expected by the sheep. Acoustic intensities, *e.g.*, I_sppa_ of 23.9 W/cm^2^ or 35 W/cm^2^, much higher than one used in present study, typically do not generate any skin sensations at the scalp in humans^49,50,53^, yet, FUS has shown to elicit mild nociception at the skin in human hands^54^. Although it is difficult to ascertain the causes for different levels of tolerability, especially in the absence of thermal rise at the sonication path/target, we speculate that the variations of acoustic intensity levels at the scalp (and/or potential skin sensations) due to different sonication trajectories and skull geometries might have contributed, which warrant cautions needed for human applications. Apart from tactile sensation at the scalp, potential skull heating should not be underestimated in humans having slightly thicker skull than sheep.

The response measures (*i.e.,* response rates and EMG amplitudes) did not show dependency on the acoustic intensities used (Supplementary Fig. S5), although we initially hypothesized that a higher intensity would yield more robust responses. We conjecture that the limited available data might have played a role in this finding and future studies using a group of large animals undergoing sonication with varying acoustic intensities, in which statistical analysis of both within- and between-condition comparisons can be conducted, are needed to clarify the effects of acoustic intensity in determining responsiveness to FUS stimulations.

The mechanism behind the neuromodulatory effects and dependency of acoustic parameters have started to be revealed by the increasing number of studies through various routes, for example, investigation of neuron-level responses to exposure to the acoustic pressure^55,56^ or through numerical models^57,58^ (comprehensive lists of recent hypotheses on the potential mechanism are found elsewhere^59^). Cell type-specific stimulation of excitatory/inhibitory neurons (*e.g.*, excitatory regular spiking pyramidal neurons versus inhibitory fast spiking interneurons), which is dependent on acoustic parameters, has been suggested as a contributing mechanism behind the bimodal effects^60^. Involvement of calcium channel-mediated astrocytes activity has also been implicated^61^. The underlying mechanism for these bimodal effects, perhaps ramified from orchestrations of different cell-type responses and their tissue-level responses, remains to be a subject of further investigation.

### Post-sonication behavior monitoring and histological assessment

The safety of FUS applications in awake sheep was evaluated based on behavioral observation during/after sonication followed by histological analysis. Since we used 71.4% and 50.0% shorter total sonication durations at comparable intensities to those used in our previous study on anesthetized sheep^10^ in which the sonication did not generate detectable temperature change of the tissue, temperature elevation is not likely in the present study. None of the sheep showed abnormal behavior during and after the sonication sessions, and no signs of brain tissue damage was observed from the histological analysis (Fig. 7). In spite of the use of higher I_sppa_ compared to our previous study that yielded the observation of a single incident of micro-hemorrhage^11^, we did not find any evidence that may indicate deleterious effects from the sonication, which is in line with a recent histologic safety study using sheep^62^. These results indicate that the neuromodulatory FUS was safely administered to awake sheep with the sonication parameters (*e.g.*, 200-ms sonication with inter-stimulus interval of 5 s for excitation and prolonged exposure to 1-min sonication for suppression) through multiple days of FUS sessions (at least 24-h interval), adding to previous safety data on anesthetized large animal models of sheep^10^ and swine^7^, as well as non-human primates^62^. In addition, this study brings important implications assuring that stimulatory effects can be achieved safely at intensities clearly above the acoustic intensity, *e.g*., 20 times higher (14.4 W/cm^2^ I_spta_) than the regulatory limit for most ultrasound imaging devices (720 mW/cm^2^; a comprehensive review of acoustic parameters used in FUS-mediated neuromodulation was described in Lee et al*.* ^63^).

### Conclusions

We established an awake animal model of sheep with FUS headgear, which enables investigations on non-invasive and non-pharmacological modulation of FUS-induced cortical and subcortical excitability without confounding effects from anesthesia. To our knowledge, this is the first study to demonstrate the bimodal effects of FUS in a freely moving large animal model. The ability to use neuromodulatory FUS in awake animal models will enable investigations that cannot be conducted with anesthesia such as studies on disease models that are sensitive to anesthetic states (*e.g.*, epilepsy). FUS neuromodulation may offer a new mode of neurotherapeutics for the treatment of neurological and psychiatric conditions. For instance, excitatory sonication can be used for enhancing motor function for stroke patients^64^ and treating depression^65^. Suppressive sonication may be useful for stabilizing abnormal hyper-excitability of the brain, such as in epilepsy^2^, or for taming stress-induced anxiety^66^. Further investigation on lasting neuromodulatory effects after sonication and potential changes neural plasticity are crucial to verify the therapeutic effects of FUS.

## Methods

### Animals and acclimation/training

Animal experiments were conducted under the approval and according to guidelines and regulations set forth by the Institutional Animal Care and Use Committee of the Brigham and Women’s Hospital. The present study was carried out in compliance with the ARRIVE guidelines (https://arriveguidelines.org/). Only ewes (Polypay; age = 24.5 ± 5.3 weeks; weight = 43.9 ± 5.4 kg, mean ± s.d., *N* = 10) were used because males may grow scurs that can excessively attenuate/distort sonication. There were no restraints on daily food/water intake other than those needed for the imaging procedure. We used a fabric harness vest (TG-526, OneTigris, Shenzhen, China) to guide animals during experimental procedures and to attach a telemetry EMG device (Fig. 1e). All sheep were incrementally acclimated to wearing the vest prior to experimental procedures. In addition, while wearing a headgear with a mock-up transducer, the animals received training to walk on a treadmill (Cadence G 5.9i, Weslo, Logan, UT) for a minimum of 5 min at a comfortable stride speed of 0.5–1.2 mph (0.2–0.5 m/s). Once fully acclimated and trained, animals were given additional training sessions intermittently throughout the study period.

### Wearable FUS headgear

The ring-shaped plastic headgear frame was designed using CAD software (Solidworks Crop., Concord, MA) and manufactured using a 3D printer (Form2; Clear V4 plastic resins, FormLabs Inc., Somerville, MA). The FUS transducer was connected to the frame via an articulating arm (MA207, Ikan, Houston, TX) which enabled maneuvering of the transducer to a desired position and orientation. The frame had three support legs that were placed over the sheep’s head, and their surfaces were angled to make comfortable contact with the scalp. For repeated positioning of the frame across multiple FUS sessions, each of three support legs contained a 2-mm diameter groove that was aligned with tattoo markings on the scalp (Fig. 1c,d). Two additional blunt Nylon screws (M8 size) were located rostrally to make a contact with the scalp, providing added support. Donut-shaped MR fiducial markers (PinPoint, Beekley Crop., Bristol, CT) were attached on the headgear for image-registration between the virtual MR space and the real space. The assembled headgear weighed 440 g.

### MRI data acquisition and image-guidance for FUS targeting

For MRI data acquisition, food/water intake was temporarily restricted (hay/food pellets/water = 48 h/24 h/6 h). Sheep were sedated by intramuscular injection of xylazine (0.1 mg/kg) followed by tiletamine/zolazepam (Telazol, 2–4 mg/kg) and intubated under the monitoring of end-tidal carbon dioxide, peripheral oxygen saturation, and heart rate. Additional doses of intravenous Telazol (0.5–0.75 mL every 15–20 min) were given as needed. After shearing/shaving the wool over the sheep scalp, the headgear frame was placed over the scalp and three fiducial points were tattooed using an insulin syringe (~ 1 mm in diameter; one on the snout, midline rostral to eyes and two on the skin medial to the ears; Fig. 1c).

Sheep were then positioned into a 3 Tesla MRI Siemens scanner (Skyra, Munich, Germany). A head/neck 20 coil was used to acquire MRI data, and foam paddings were applied around the head to minimize head movement. The 3D magnetization-prepared rapid gradient-echo (MP-RAGE) pulse sequence was used to acquire an anatomical image covering the entire head. The standard gradient-echo echo-planar-imaging (EPI) pulse sequence was used for functional MRI (fMRI) acquisition covering the brain. During EPI data acquisition, passive tactile stimulations were given with gentle mechanical squeeze to the right hind limb muscle (around 2 Hz) of the sheep to activate motor circuit including the left M1 cortex as well as the corresponding thalamic area^10,11,22–24^. The stimulations were given for 20 s three times interleaved by four 20-s resting (non-stimulus) periods. Additional T1-/T2-weighted anatomical images were obtained from the same field-of-view as the EPI acquisition and used for registration between EPI and MP-RAGE images. The detailed MRI acquisition protocol is described in *Supplementary Methods* (see “*MRI acquisition*”). The fMRI data were preprocessed with motion correction (*i.e.*, realignment), followed by spatial smoothing (6 × 6 × 6 mm^3^) using the SPM8 software (Wellcome Department of Imaging Neuroscience, University College London, London, UK; www.fil.ion.ucl.ac.uk/spm). A functional activation map specific to the stimulation blocks was estimated using the general linear model with a task-specific canonical hemodynamic response and visualized with a statistical threshold of uncorrected *P* < 0.05. After the MRI acquisition and data processing, a sheep skull model was used as a mechanical platform on which a custom-fit headgear was placed, and an in-house neuro-navigation system^13,21^ was used for planning and targeting sonication. As the incidence angle (the angle between sonication directional vector to the skull surface) has been shown to be important in providing sub-millimeter spatial accuracy in sonication of sheep^13^, we kept the incident sonication direction as perpendicular as possible to the skull surface (within 10°).

### Actuation of FUS transducer and EMG data acquisition

A single-element FUS transducer (GPS200-D40-FL57-MR, Ultran Group, State College, PA) with a focal length of 30 mm from its exit plane was operated at a fundamental frequency of 250 kHz. The piezo-element disc of the transducer in 43 mm diameter was coupled with an acoustic lens of 17 mm radius-of-curvature. Two serially-connected function generators (33500B, Keysight, Santa Rosa, CA) generated an electrical waveform (Fig. 1f), which was amplified by a linear power amplifier (240L, Electronics and Innovations, Rochester, NY) to actuate the FUS transducer with impedance matching (JT-800, Electronics and Innovations) in-between. The acoustic intensity profile along the longitudinal (30 × 70 mm^2^ with 1 mm step) and transverse planes (30 × 30 mm^2^ with 1 mm step) was estimated in a degassed water tank using a needle-type hydrophone (HNC200, Onda, Sunnyvale, CA) installed on a three-axis robotic stage (Bi-Slides, Velmex, Bloomfield, NY). The detailed methods of the transducer characterization can be found elsewhere^10^.

Prior to the sonication experiments, the wool of the sheep was clipped and shaved over the lower back (10 × 15 cm^2^; ~ 20 cm rostral from the tail), the gastrocnemii of the hind limbs, and the scalp. After the shaving, the harness vest was placed over the animal, and the telemetry EMG transmitter/preamplifier setup (analog to digital converter with 16 bits and 256 Hz/channel; gMOBIlab + , g.tec, neurotechnology, Albany, NY) was attached to the vest. Four surface cup electrodes (g.LADYbird, g.tec, neurotechnology) were filled with conductive gel and attached to the gastrocnemii of both hind limbs while the reference and the ground electrodes were attached on the lower back. Elastic bandaging tape (Vetrap, 3 M, Saint Paul, MN) was used to keep all electrodes secured in place while the animal was moving freely (Fig. 1e).

After preparing the EMG setup, the FUS transducer mounted on the headgear was coupled with a compressible polyvinyl alcohol (PVA) hydrogel manufactured in-house (341,584, Sigma-Aldrich, St. Lousi, MO; 9% weight per volume in degassed water, two freeze–thaw cycles) for acoustic coupling^67^. A generic ultrasound gel was also applied to all the interfaces. The thicknesses of the PVA hydrogels for the M1 and thalamus stimulations were 25 mm and 5 mm from the exit plane of the transducer and these PVA hydrogels were compressible up to 19 mm and 4 mm, respectively. Thus, the acoustic focus (30 mm focal depth) can reach each brain region either around 5–11 mm or 25–29 mm deep from the scalp. Once the headgear was put on the sheep’s head in alignment with the tattooed points, it was secured in place by a chin strap. An additional elastic bandaging tape was also wrapped over the headgear to further secure the position of the headgear (Fig. 1d). Care was given not to block the eyes during the headgear placement.

For excitatory sessions, the power cable to the transducer was connected to a swivel connector (FMRJ1002, Fairview Microwave Inc., Lewisville, TX) over the pen, which allowed for free movements of sheep within a limited range. In suppressive FUS sessions, a treadmill was set up in a space next to the animal pen and the sheep voluntarily walked/strolled on a treadmill at a constant gait speed (0.5–0.8 mph; fixed speed to accommodate different gait style) for at least 2 min before commencing the data acquisition. The power cable was held by an experimenter, instead of being connected to the swivel connector, to provide wider range of movement than in the excitatory session.

### Preparation of EMG data and derivation of steps taken during suppressive sonication

The acquired EMG data were preprocessed using MATLAB R2019b (Mathworks, Natick, MA). First, signal noises including cardiorespiratory signal fluctuation were reduced using a high-pass filter of 20 Hz (via “highpass” MATLAB function with the steepness parameter of 0.95)^68^ and 60 Hz notch-filter. Then, full-wave rectification and Gaussian smoothing (with a temporal window of 44 ms) were applied^18^.

The number of steps taken per time segment, measured during treadmill walking, was computed from both hind limbs using MATLAB’s ‘findpeaks’ algorithm based on the preprocessed EMG signals. The video data were also cross-referenced to validate the number of steps. The averaged values of the number of steps from each hind limb were then calculated and compared across experimental conditions. For the ANOVA analysis, the missing data due to data exclusion were substituted using the “Series mean” approach in the SPSS software (IBM, Amrok, NY).

### Post-sonication behavioral monitoring and histology

Post-sonication behavior and body condition of the animal were regularly monitored (one to three days between the monitoring). Sheep were euthanized through intravenous injection of pentobarbital sodium and phenytoin sodium solution (Euthasol,100 mg/kg) after completing their last sonication session with different time intervals (*N* = 4 within 24 h, *N* = 2 for 2 weeks, and *N* = 4 for 2 months). The sedation procedure that was used for the MRI acquisition was performed prior to euthanasia. For the histology analysis, H&E (GHS-2–16, Sigma-Aldrich), VAF-toluidine blue staining (A3908, Sigma-Aldrich), IHC of GFAP (ab7260, Abcam, Cambridge, UK) and caspase-3 staining (ab4051, Abcam) were conducted on the brain tissues harvested from the M1 area (within ~ 5 mm proximity to the adjacent skull), the thalamus, and unsonicated brain tissue from the right hemisphere opposite to the sonication (as a control site). The detailed procedure for tissue extraction and histological assay followed our previous study^10^.

## Supplementary Information

Supplementary Information.
