# Supplementary material for: Transcranial focused ultrasound modulates cortical and thalamic motor activity in awake sheep
Source: Sci Rep. 2021 Sep 29;11:19274. doi: 10.1038/s41598-021-98920-x (PMC8481295; doi:10.1038/s41598-021-98920-x)
Supplement: Supplementary file 1 — Supplementary Information. [file 41598_2021_98920_MOESM1_ESM.pdf]

Supplementary Information

Transcranial focused ultrasound modulates cortical and thalamic motor activity in awake sheep

Hyun-Chul Kim[1], Wonhye Lee[1], Jennifer Kunes[1], Kyungho Yoon[1], Ji Eun Lee[1], Lori Foley[2], Kavin Kowsari[3], Seung-Schik Yoo[1]

[1]Department of Radiology, Brigham and Women's Hospital, Harvard Medical School, Boston, MA, USA

[2]Translational Discovery Laboratory, Brigham and Women's Hospital, Boston, MA, USA

[3]Department of Mechanical Engineering, Massachusetts Institute of Technology, Cambridge, MA, USA

Supplementary Methods

MRI acquisition

The 3D magnetization-prepared rapid gradient-echo (MP-RAGE) pulse sequence was used to acquire an anatomical image covering the entire head (repetition time [TR]/echo time [TE] = 2,300/2.3 ms; field-of-view [FoV] = 250 × 250 mm^2; voxel size = 0.98 × 0.98 × 1.0 mm^3 with no gaps between slices; flip angle [FA] = 8 °; 160 sagittal slices). The gradient-echo echo-planar-imaging (EPI) pulse sequence was used for functional magnetic resonance imaging acquisition covering the entire brain (TR/TE = 2,000/40 ms; FoV = 180 × 180 mm^2; voxel size = 2.81 × 2.81 × 3.0 mm^3 with no gaps; FA = 90 °; 20 axial slices). Additional T1-weighted (TR/TE = 400/9.4 ms; FoV = 180 × 180 mm^2; voxel size = 0.35 × 0.35 × 3.0 mm^3 with 0.6 mm slice gap; FA = 65 °; 20 axial slices) and T2-weighted anatomical images (TR/TE = 3,940/100 ms; FoV = 146.3 × 180 mm^2; voxel size = 0.35 × 0.35 × 3.0 mm^3 with 0.6 mm slice gap; FA = 150 °; 20 axial slices) were obtained from the same FoV as the EPI acquisition and were used for registration between EPI and MP-RAGE images.

Evaluation of thermal effects

We examined the potential thermal effects from sonication by estimating the temperature increase at the sonicated M1 and adjacent skull by sequentially solving the Khokhlov-Zabolotskaya-Kuznetsov (KZK) equation and bio-heat transfer equation through an open-source high intensity FUS (HIFU) simulator based on MATLAB scripts[32]. The simulation was performed at resolution of 0.5 mm based on the previous numerical study of FUS propagation through the skull[13] using the sonication parameters and the maximum *in situ* acoustic intensity for each of excitatory (20.5 W/cm^2 I_{sppa}) and suppressive sonication (13.7 W/cm^2 I_{sppa}). The simulation of temperature rise from the M1 was conducted with a temporal resolution of 0.2 ms using the acoustic properties (speed of sound of 1482 m/s, density of 1000 kg/m^3, attenuation coefficient of 0.217 dB/m·MHz^{-1}) and thermal properties of the brain (specific heat of 3696 J/kg·K^{-1}, thermal conductivity of 0.55 W/m·K^{-1}, perfusion rate of 14.1 kg/m^3·s^{-1})[7]. For the simulation of thermal effects in the skull

adjacent to the M1, specific heat of 1300 J/kg·K^{-1}, thermal conductivity of 0.4 W/m·K^{-1}, and perfusion rate of 0.143 kg/m^{3}·s^{-1} were used[66-68].

Supplementary Results

Excitatory sonication

Comparison of EMG amplitudes between on-target and off-target conditions

The group-averaged amplitudes of the EMG signals from each of experimental conditions (on-target, off-target and no-FUS) were compared using one-way ANOVA followed by Tukey-Kramer *post-hoc* analysis. In the comparison between on-target and off-target condition, the group-averaged amplitudes of the EMG signals from the hind limb contralateral to sonication became significantly greater upon on-target stimulation to the M1 and thalamus, compared to those obtained from the off-target condition (one-way ANOVA followed by Tukey-Kramer *post-hoc* analysis, $F(2,27) = 14.2$–36.5 for the M1 and $F(2,27) = 9.0$–34.9 for the thalamus, $P < 0.001$ presented by green dots in **Figs. S2a** and **b**). The increased EMG amplitude was found in the time segments of 50.0–57.8 and 73.4–93.0 ms in the M1 stimulation. During the thalamic stimulation, greater EMG amplitude from the hind limb contralateral to sonication was found in the time segments of 57.8–89.1 and 475.8–479.7 ms. These results indicate that the on-target sonication of the motor circuits selectively increased the EMG amplitudes from the hind limb contralateral to the FUS. In the case of the left hind limb (ipsilateral to sonication), none of the corresponding comparison pairs across conditions showed statistical differences ($P > 0.05$, $N = 10$; **Figs. S2c** and **d**).

Comparison of EMG amplitudes between on-/off-target and no-FUS conditions

The EMG signal from the right hind limb contralateral to on-target sonication to the M1 was significantly greater than that from the same leg of the no-FUS condition within time segments of 42.0–108.6, 128.1–143.7, 155.5–186.7, 214.1–218.0, and 714.1–725.8 ms after the FUS onset (one-way ANOVA followed by Tukey-Kramer *post-hoc* analysis, $F(2,27) = 9.1$–36.5, $P < 0.001$ presented by brown dots in **Fig. S2a**). In the thalamic stimulation, a greater EMG signal from the right hind limb compared to that in the no-FUS condition was observed in time segments of 50.0–93.0, 124.2–128.1, 175.0–182.8, 210.2–237.5 and 479.7 ms ($F(2,27) = 9.3$–34.9, $P < 0.001$ presented by brown dots in **Fig. S2b**). Between off-target and no-FUS

conditions, no difference in the EMG amplitude was found from the right hind limb ($P > 0.05$; **Figs. S2a** and **b**). There were no statistical differences in EMG amplitudes between on-/off-target and no-FUS conditions from the left hind limb ($P > 0.05$; **Figs. S2c** and **d**).

Suppressive sonication

Hind-limb specific comparisons across the experimental conditions in each time segment

The EMG amplitudes of the right hind limb, contralateral to sonication, were significantly decreased during the F1 segment (-7.6 ± 7.1%) from sonication of the M1, compared to those from the on-target thalamus (-2.4 ± 2.5%), off-target (0.1 ± 2.3%) and no-FUS (0.6 ± 2.2%) conditions (one-way ANOVA, $F(3,36) = 8.5$, $P < 0.05$, followed by Tukey-Kramer *post-hoc* analysis, $P < 0.05$ presented by black brackets in **Fig. S4a**). The significant reduction of the contralateral EMG amplitude was found in the F2 (-3.5 ± 2.6%) segment with respect to the off-target (-0.2 ± 1.4%) and no-FUS (-0.5 ± 2.2%) conditions ($F(3,36) = 7.0$, Tukey-Kramer *post-hoc* analysis, $P < 0.05$). Also, the suppressive sonication of the M1 significantly reduced the contralateral EMG amplitude during the P3 segment (-2.7 ± 1.8%), compared to the amplitude from the off-target (0.3 ± 2.9%) condition ($F(3,36) = 3.0$, Tukey-Kramer *post-hoc* analysis, $P < 0.05$). Other than P3, no differences were found during the post-sonication segments.

Suppressive sonication of the thalamus selectively reduced the EMG amplitudes from the right hind limb in the F2 (-4.1 ± 3.2%; Tukey-Kramer *post-hoc* analysis, $P < 0.01$), compared to those from the off-target and no-FUS conditions (**Fig. S4a)**. No differences were observed in all other time segments. There was no significant difference in the EMG amplitude obtained from the left hind limb (ipsilateral to sonication) across time segments throughout the experimental conditions (**Fig. S4b)**.

Supplementary Table

Animal ID	Suppressive FUS to the M1			Suppressive FUS to the Thalamus		
	Suppressive Effects		Successful Response	Suppressive Effects		Successful Response
	'F1'	'F2'		'F1'	'F2'	
1	Y	N	Y	N	Y	Y
2	N	N	N	N	Y	Y
3	N	Y	Y	Y	Y	Y
4	Y	Y	Y	Y	N	Y
5	N	Y	Y	N	Y	Y
6	Y	Y	Y	N	N	N
7	N	N	N	N	Y	Y
8	Y	Y	Y	Y	Y	Y
9	N	N	N	Y	N	Y
10	Y	N	Y	N	N	N

Table S1. Sheep-specific responsiveness to suppressive sonication applied to either M1 or thalamus. Presence of successful effects was determined if sheep showed at least one responsiveness (*i.e.*, 'Y') in either 'F1' or 'F2' segment (*i.e.*, duration of suppressive sonication). 'N' indicates the absence of responsiveness during suppressive sonication.

Supplementary Figures

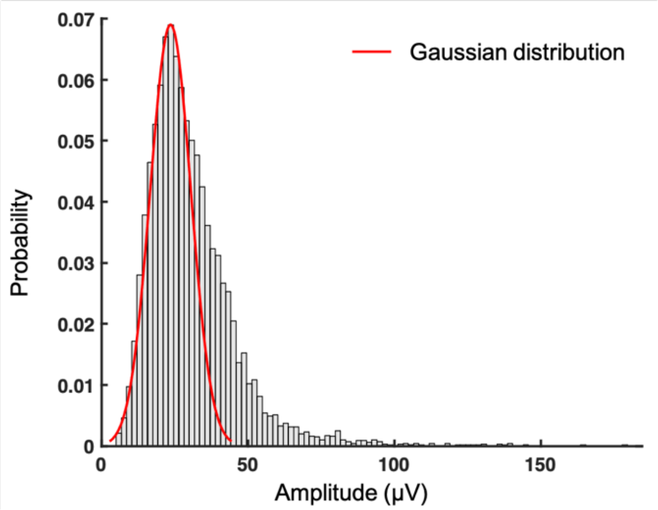

Figure S1. **Exemplar positively-skewed distribution of EMG amplitudes obtained from one of the sheep during pre-FUS segments**. The red line indicates a Gaussian distribution fitted to the EMG amplitude profile. The mode value of the EMG amplitude distribution was set to the mean value of the Gaussian distribution.

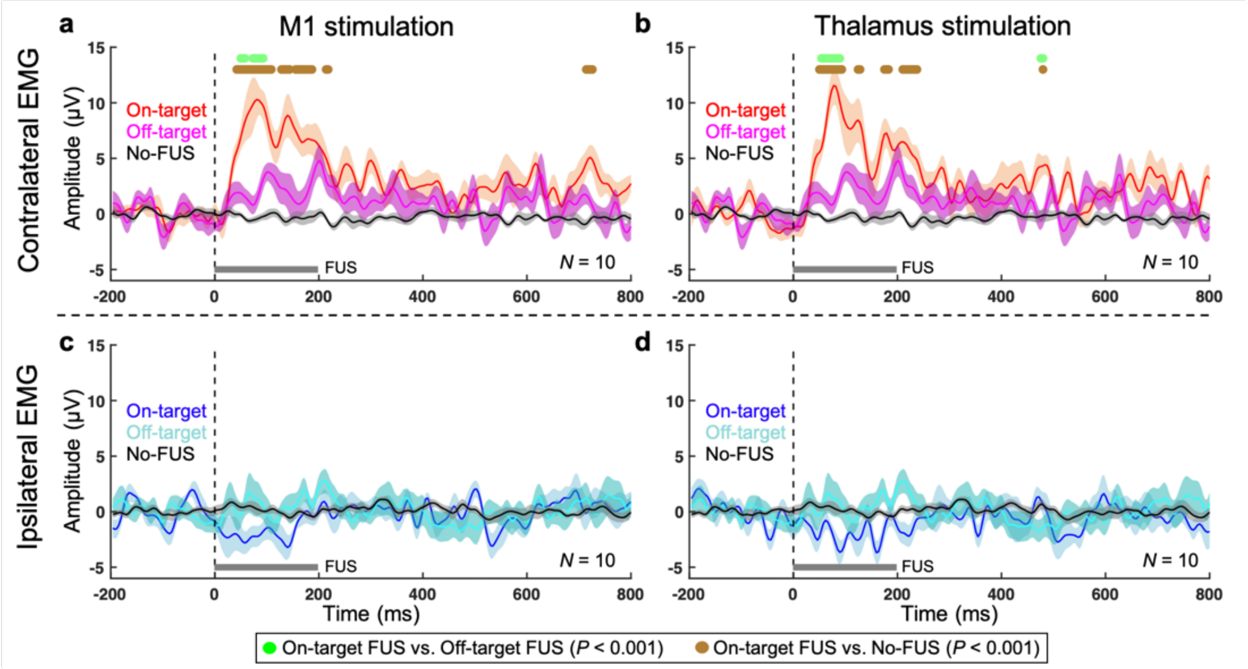

Figure S2. Comparison of time-locked EMG measurements from the excitatory sonication among experimental conditions. Comparisons of group-averaged EMG amplitudes ($N = 10$) in excitatory FUS to the M1 (**a**, **c**) and the thalamus (**b**, **d**) with respect to the EMG amplitudes from the off-target and no-FUS conditions. The red and magenta lines represent EMG amplitudes from the hind limb contralateral to sonication while the blue and cyan lines represent EMG amplitudes from the ipsilateral hind limb. Black lines represent EMG obtained during the no-FUS condition. The shaded area indicates the standard errors across all animals. The gray bar is the duration of sonication (200 ms). The green (for on-target FUS vs. off-target FUS) and brown (for on-target FUS vs. no-FUS) dots indicate significant differences (one-way ANOVA followed by Tukey-Kramer *post-hoc* analysis, $P < 0.001$) in the EMG amplitudes between the paired conditions.

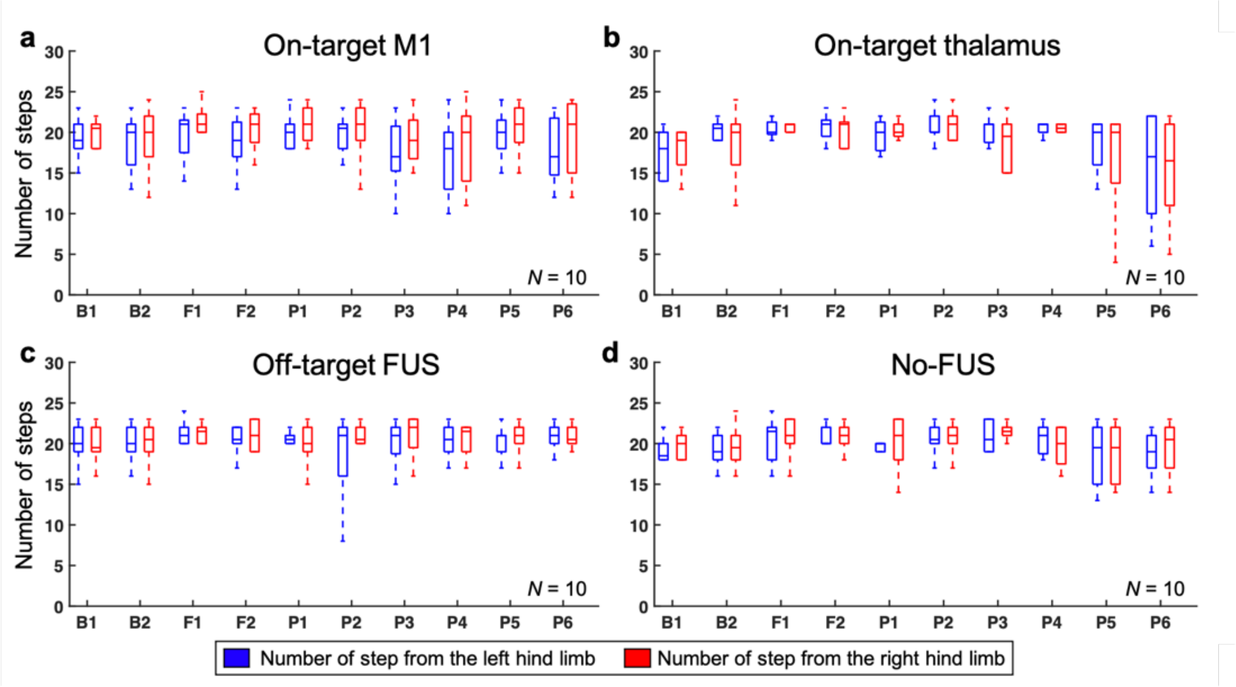

Figure S3. Behavioral gait patterns in suppressive sonication sessions. The number of steps for each time segment ('B1'–'P6') taken from both hind limbs in (**a**) on-target M1, (**b**) on-target thalamus, (**c**) off-target FUS and (**d**) no-FUS conditions during suppressive FUS experiments, represented with boxplots (the bottom and top edges of the box indicate the 25th and 75th quartiles, respectively; horizontal lines: the median; whiskers: the minimum or maximum value, $N = 10$ sheep). The blue boxplots indicate the number of steps obtained from the left hind limb while the red boxplots indicate the number from the right hind limb (contralateral to the sonication). There were no significant differences in the number of steps taken from hind limbs across the experimental conditions (one-way ANOVA; $P > 0.05$). Also, no significant time-dependent (one-way repeated measures ANOVA; $P > 0.05$, across 'B1'–'P6') or limb-specific (paired *t*-test, $P > 0.05$, left vs. right hind limbs) trends in the number of steps taken were found.

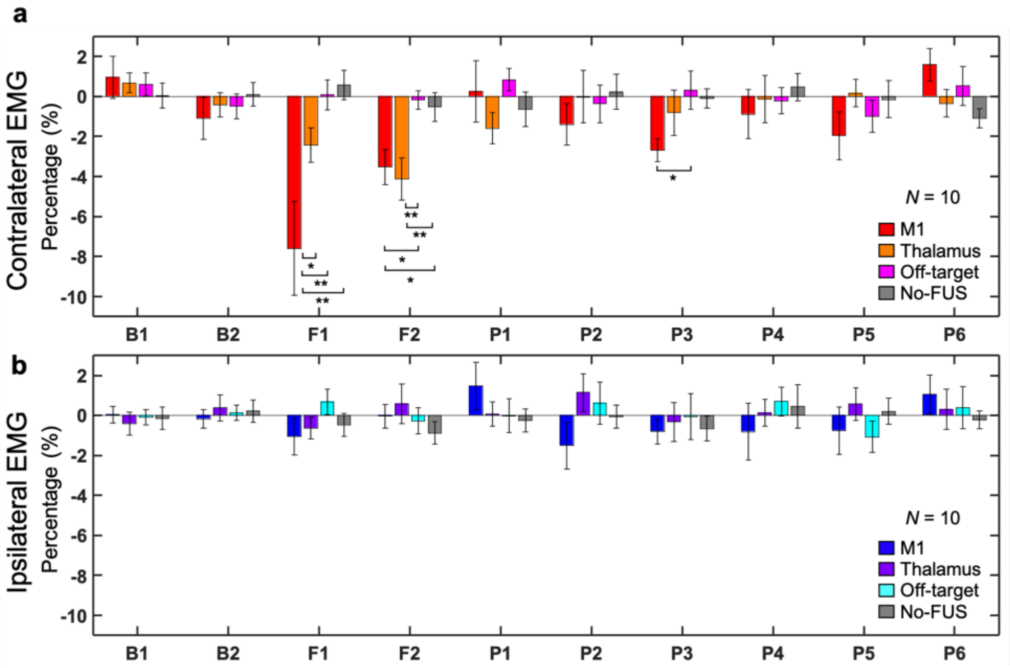

Figure S4. Hind-limb specific comparisons across the experimental conditions in each time segment of B1–P6. Percentage differences in the EMG amplitudes (**a**) contralateral and (**b**) ipsilateral to suppressive FUS sonication among experimental conditions (N = 10 sheep). The condition-dependent percentage difference in the amplitudes was compared using one-way ANOVA followed by Tukey-Kramer *post-hoc* analysis ($^{*}P < 0.05$, $^{**}P < 0.01$). The red, orange, magenta, and gray color bars indicate average percentage values of the contralateral EMG signals in the on-target M1 & thalamus, off-target, and no-FUS conditions, respectively. The blue, purple, cyan, and gray color bars indicate average percentage values of the ipsilateral EMG signals. The error bars indicate standard errors.

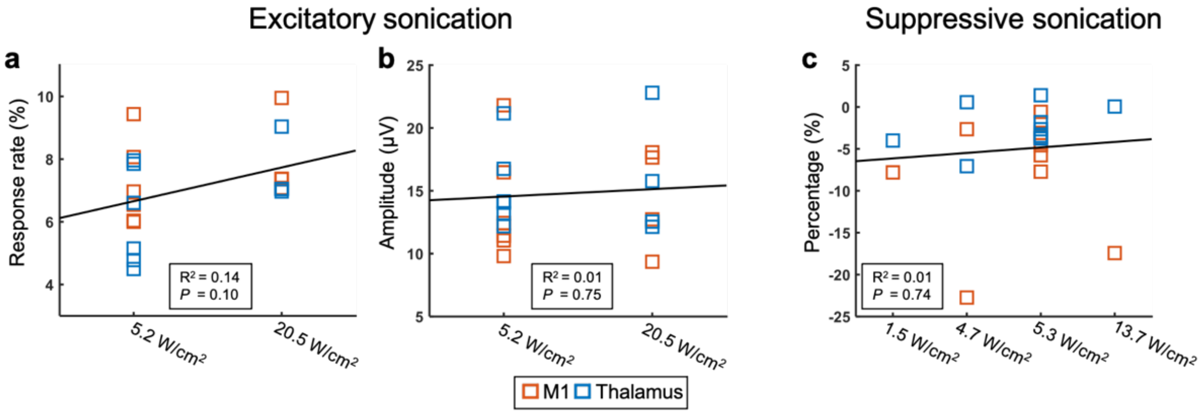

Figure S5. The relationship between *in situ* I_{sppa} and response rates or EMG signals. Scatter plots of *in situ* I_{sppa} and corresponding (**a**) response rates and (**b**) maximum EMG amplitude during FUS segment from excitatory sonication, as well as (**c**) percentage change of EMG signal amplitude from suppressive sonication. R^2 is a regression coefficient. *P* is a *p*-value estimated from regression analysis.

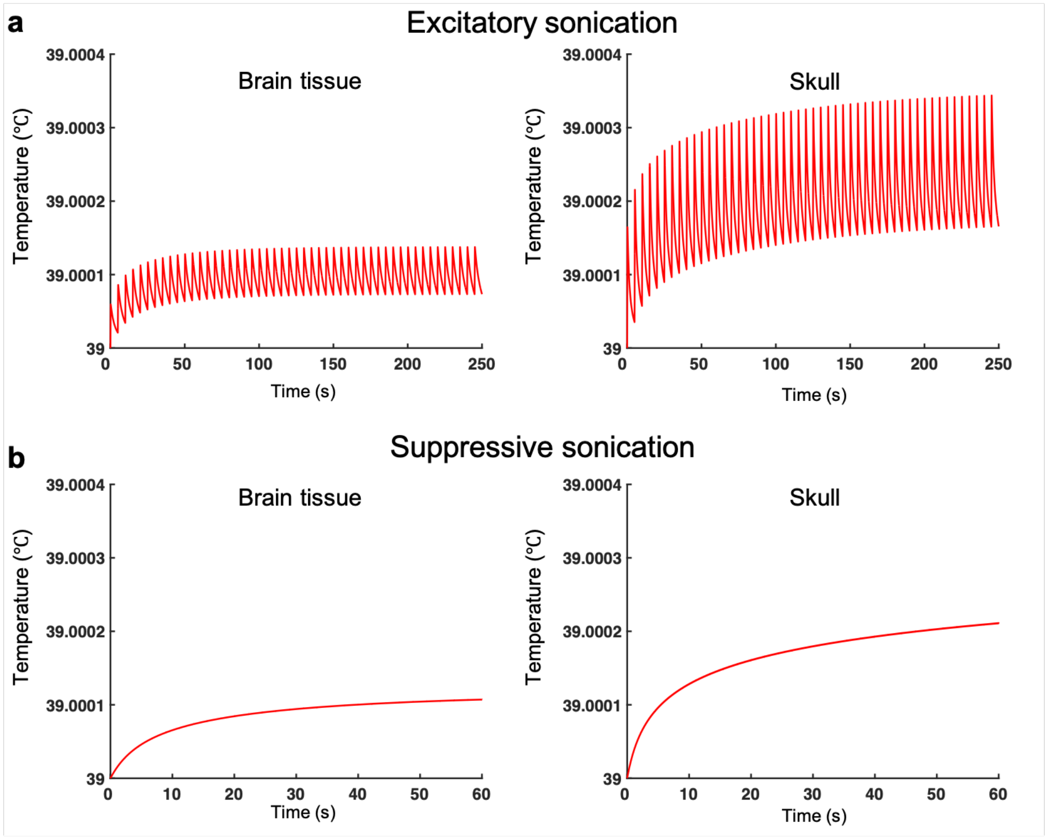

Figure S6. Estimated temperature rise at the sonicated M1 (brain tissue) and adjacent skull for (**a**) excitatory sonication given at *in situ* I_{sppa} = 20.5 W/cm^2 with 50 repetitions every 5 s and (**b**) suppressive sonication given for 1 min at *in situ* I_{sppa} = 13.7 W/cm^2.
